# Dapagliflozin induces renal lipidomic remodeling and systemic metabolic improvement

**DOI:** 10.1186/s13062-026-00800-9

**Published:** 2026-04-17

**Authors:** Anni Li, Yuxuan Ye, Jinlin Tang, Jiawei Hu, Min Shi, Yangyang Wu, Chenbo Ji, Hong Zhang

**Affiliations:** 1 Huai’an Institute of Metabolic Diseases of Nanjing Medical University, Huai’an, 223300 China; 2 Nanjing Women and Children’s Healthcare Institute, Women’s Hospital of Nanjing Medical University, Nanjing Women and Children’s Healthcare Hospital, Nanjing, Jiangsu China; 3 The Affiliated Huai’an No. 1 People’s Hospital of Nanjing Medical University, Huai’an, 223300 China

**Keywords:** Dapagliflozin, Lipidomics, Spatial metabolomics, DN

## Abstract

**Background:**

Diabetic nephropathy (DN) is a leading cause of end-stage renal disease worldwide. Sodium-glucose cotransporter-2 inhibitors (SGLT2is), such as dapagliflozin, have demonstrated renoprotective effects in the treatment of DN. In addition to kidney protection, SGLT2is confer significant cardiovascular and hepatic benefits; however, the mechanisms underlying these multi-organ protective effects have not yet been fully elucidated.

**Methods:**

A total of 51 patients with newly diagnosed DN were enrolled and received dapagliflozin treatment (10 mg/day; AstraZeneca) for 12 weeks. Circulating lipid profiles were analyzed using untargeted lipidomics. To further characterize renal lipid alterations, spatial metabolomics was performed on kidney tissues obtained from dapagliflozin-treated *db/db* mice.

**Results:**

Dapagliflozin treatment significantly reduced serum triglyceride levels while increasing high-density lipoprotein cholesterol in DN patients. Untargeted lipidomic analysis revealed extensive remodeling of the circulating lipidome, marked by reductions in pro-fibrotic and pro-inflammatory lipid species and concurrent increases in renoprotective lipids, including fatty acid esters of hydroxy fatty acids. Machine-learning analyses identified specific lipid ratio changes that were positively correlated with renal function parameters, notably ratios involving SM(d14:0/30:1) and PC(16:0e/18:2). Furthermore, spatial metabolomic profiling in db/db mice demonstrated that dapagliflozin alleviated renal lipotoxicity by reducing the accumulation of toxic lipid species and promoting lipid redistribution predominantly within the renal cortex. Mechanistically, dapagliflozin treatment was associated with enhanced renal fatty acid β-oxidation and sphingolipid degradation while suppressing key anabolic pathways, including de novo lipogenesis and glycerophospholipid and sphingolipid biosynthesis. These metabolic alterations were further evidenced by altered expression of key regulatory enzymes.

**Conclusion:**

Dapagliflozin is associated with remodeling of renal lipid metabolism in DN, accompanied by improvements in systemic lipid profile. The improvement in circulating dyslipidemia may partially explain the cardiovascular and hepatic protective effects associated with dapagliflozin therapy. Collectively, these findings provide mechanistic insight into the lipid-mediated, multi-organ benefits of dapagliflozin in DN.

**Graphical Abstract:**

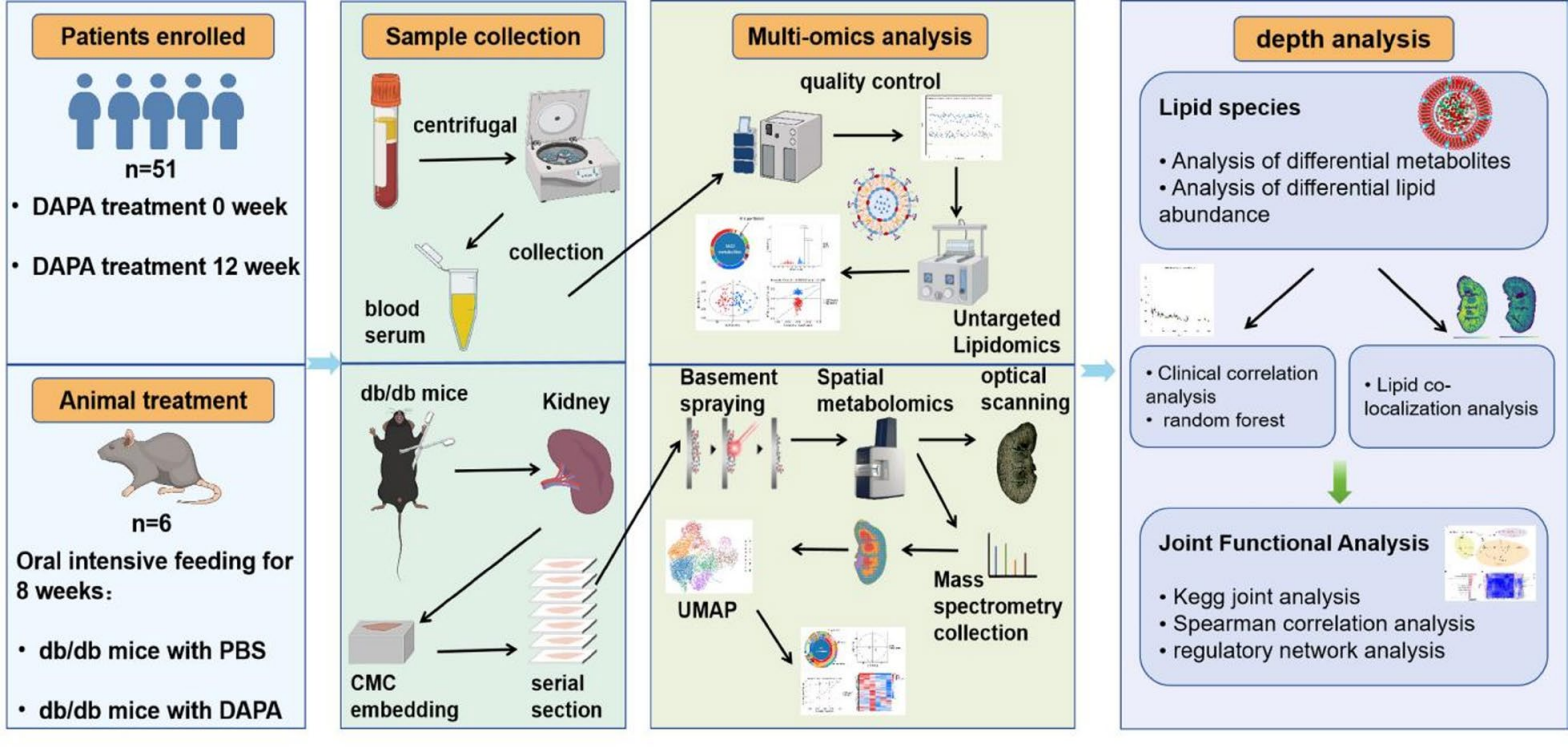

**Supplementary Information:**

The online version contains supplementary material available at 10.1186/s13062-026-00800-9.

## Introduction

Diabetic nephropathy (DN), a major microvascular complication of diabetes mellitus, is a leading cause of end-stage renal disease worldwide [1, 2]. It is estimated that approximately 20–40% of patients with diabetes develop DKD [3]. Globally, DKD accounts for 30–50% of incident ESRD cases, with proportions exceeding 50% in some developed countries [4]. Moreover, patients with DKD face a significantly higher risk of cardiovascular mortality and all-cause mortality compared to diabetic individuals without kidney involvement. Its rapidly increasing prevalence, coupled with the limited availability of effective therapeutic options [5, 6], imposes a substantial burden on both affected individuals and healthcare systems [7, 8]. Despite advances in glycemic management, the renoprotective effects of several conventional classes of antidiabetic agents remain inconsistent or modest [9]. This unmet therapeutic need has shifted research focus toward sodium-glucose cotransporter 2 inhibitors (SGLT2is), which have demonstrated robust and reproducible renoprotective effects in both preclinical models and clinical trials [10, 11].

SGLT2is are a class of antihyperglycemic agents that reduce blood glucose levels by blocking glucose reabsorption in the proximal renal tubules [12–14]. Beyond glycemic control, SGLT2is have garnered considerable attention for their pleiotropic effects, including well-documented cardiovascular and hepatic benefits [15–20]. Collectively, these observations suggest that SGLT2is exert systemic metabolic regulatory effects that extend beyond glucose lowering alone.

Among SGLT2is, dapagliflozin has been shown to slow the progression of chronic kidney disease and consistently reduce cardiovascular events in large-scale clinical trials [21–23]. Recent multicenter, randomized, placebo-controlled studies further demonstrate that dapagliflozin significantly improves hepatic steatosis and fibrosis in patients with metabolic dysfunction-associated steatohepatitis, accompanied by favorable alterations in circulating lipid profiles [24, 25]. Additional clinical evidence indicates that dapagliflozin promotes weight loss and markedly reduces perirenal fat accumulation, particularly when administered in combination with metformin [26, 27]. Notably, dapagliflozin monotherapy has also been shown to significantly decrease body fat percentage and overall body weight in patients with type 2 diabetes mellitus [28]. Collectively, accumulating evidence suggests that dapagliflozin may exert renoprotective effects through systemic and renal lipid remodeling [29–32]. These findings support the notion that dapagliflozin modulates systemic lipid homeostasis beyond its direct actions on the kidney.

In our clinical cohort of patients with DN, dapagliflozin treatment resulted in significant improvements in lipid-related clinical parameters, notably reductions in serum triglyceride levels and increases in high-density lipoprotein cholesterol (HDL-C). Despite these favorable changes, the specific lipid species involved and the mechanistic pathways through which dapagliflozin modulates lipid metabolism remain largely unresolved.

Accordingly, building on our clinical observation that dapagliflozin significantly ameliorates serum lipid accumulation in patients with DN, we performed untargeted serum lipidomic profiling to comprehensively characterize circulating lipid alterations. To further elucidate renal lipid dynamics, we conducted high-resolution spatial metabolomic analyses on kidney tissues from dapagliflozin-treated diabetic db/db mice, enabling precise mapping of intrarenal lipid distribution. By integrating lipidomic data from patients with spatial metabolomic profiles from diabetic mouse models, we identified dapagliflozin-responsive lipid species in human circulation, delineated their spatial distribution within the kidney, and elucidated the associated renoprotective metabolic pathways. Collectively, our findings provide novel mechanistic insights into dapagliflozin-mediated remodeling of the systemic lipidome and renal spatial metabolome, and offer a potential framework for identifying therapeutic targets that may facilitate precision medicine approaches and clinical translation in DN.

## Results

### Dapagliflozin ameliorates clinical metabolic parameters and remodels the serum lipidomic landscape in DN patients

We first evaluated the effects of dapagliflozin on key clinical metabolic parameters in patients with DN. As summarized in Table 1, prolonged dapagliflozin treatment resulted in significant reductions in body weight, serum triglyceride levels, and visceral fat area, while simultaneously increasing HDL-C levels. Collectively, these improvements underscore the beneficial role of dapagliflozin in regulating systemic lipid homeostasis in DN.

**Table 1:** Baseline changes of dapagliflozin in patients with diabetic nephropathy before and after treatment

Characteristic	0 week (*n* = 51)	12 weeks (*n* = 51)	*P*-value
Gender			> 0.9999
Male	35(68.63%)	35(68.63%)	
Female	16(31.37%)	16(31.37%)	
Age, years	55.58 ± 9.21	55.58 ± 9.21	> 0.9999
Weight, kg	73.17 ± 7.9	71.98 ± 8.6	0.3521
BMI, kg/m^2^	27.22 ± 2.43	27.07 ± 2.72	0.2272
Waistline, cm	95.6 ± 8.77	96.07 ± 6.83	0.9054
Hipline, cm	100.96 ± 7.6	99.52 ± 5.56	0.7912
HbA1c, %	9.89 ± 2.04****	7.39 ± 1.37	< 0.0001
CHOL, mmol/L	4.47 ± 1.33	4.33 ± 1.1	0.0906
TG, mmol/L	2.27 ± 1.67**	1.54 ± 0.92	0.0078
HDL-C, mmol/L	1.07 ± 0.25*	1.21 ± 0.23	0.0130
LDL, mg/L	2.94 ± 0.96*	2.47 ± 1.12	0.0427
FPG, mmol/L	10.4 ± 3.57****	7.85 ± 1.46	< 0.0001
eGFR, ml/min/1.73 m^2^	109.04 ± 31.24	114.71 ± 33.35	0.8223
Visceral Fat Area, cm^2^	103.17 ± 33.22	101.59 ± 38.8	0.4930

Note: Data are presented as mean ± SEM for continuous variables and n (%) for categorical variables. * *P* < 0.05, ** *P* < 0.01, *** *P* < 0.001, **** *P* < 0.0001

**Fig. 1:**
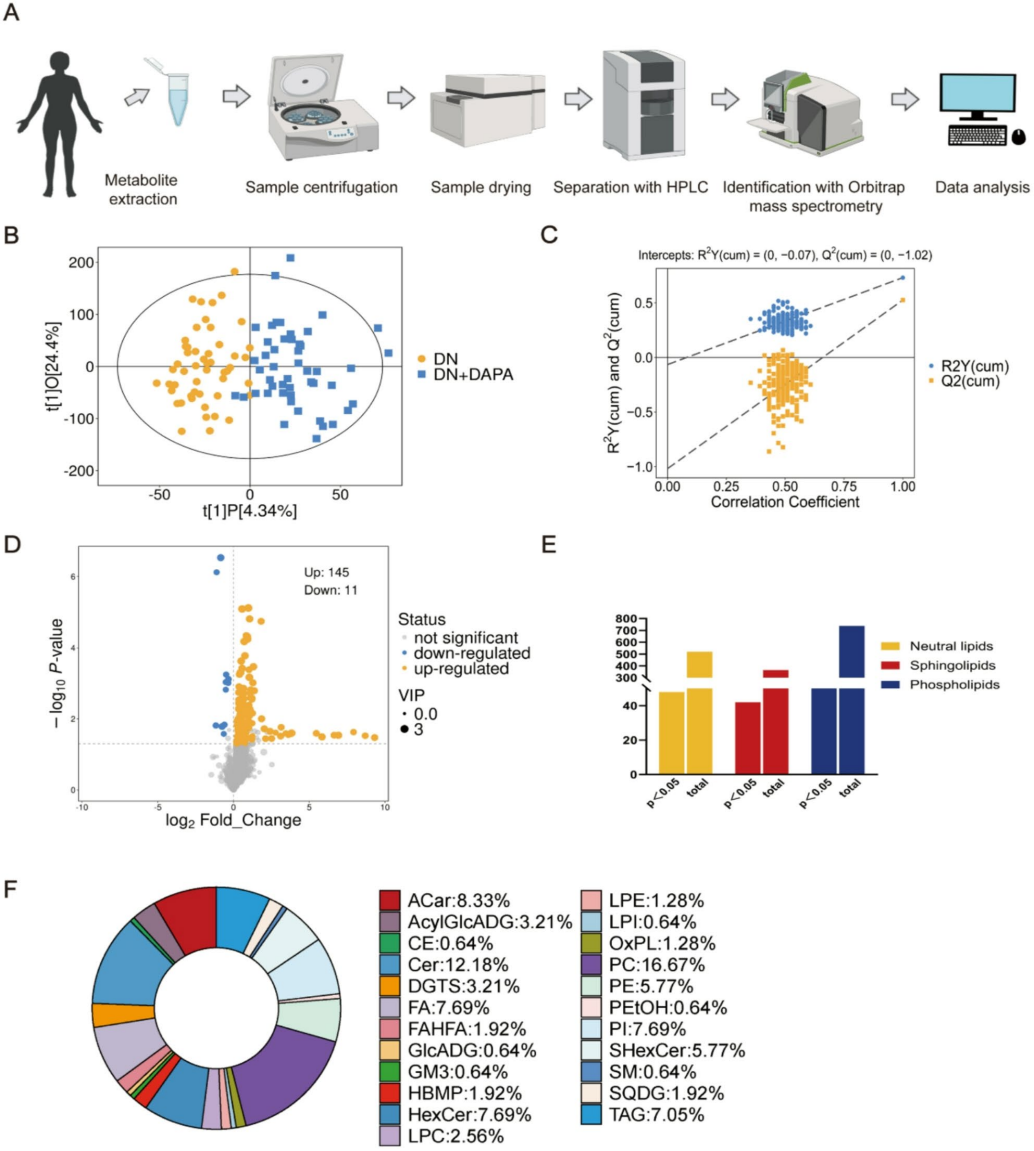
Dapagliflozin remodels the serum lipidome in diabetic nephropathy. **A**. Schematic of the liquid chromatography-mass spectrometry (LC-MS) workflow for serum lipid extraction and analysis. **B**. OPLS-DA score plots of DN group and DN+DAPA group based on LC-MS data. **C**. Statistical validation of the PLS-DA model through permutation testing. **D**. Volcano plots show differences in lipid upregulation and downregulation. **E**. **F**. The graph denotes the number of differential/non-differential abundances of lipid species. Pie charts show the differential abundance of lipid classes between two groups

To obtain deeper insight into lipid alterations at the molecular species level, we performed untargeted serum lipidomic profiling in both groups (Fig. 1A). In total, 26,987 features were initially detected; after data preprocessing and quality control, 24,690 features corresponding to 1,622 lipid species were retained for subsequent analyses (Supplementary table S1). The QC samples were analyzed to evaluate system stability, and results confirmed that the analytical system remained stable throughout the run, providing reliable data for subsequent differential analysis (Supplementary table S2). An orthogonal partial least squares discriminant analysis (OPLS-DA) model was constructed using all identified lipid features. The score plot revealed a clear separation between the DN group and the dapagliflozin-treated group (DN+DAPA), indicating distinct serum lipidomic profiles (Fig. 1B). Permutation testing confirmed the absence of model overfitting and supported the robustness of the OPLS-DA model (Fig. 1C). Collectively, these results validate the stability and reliability of the lipidomic dataset for downstream analyses.

Further, a volcano map was applied to quantify the lipids with statistical significance. Based on thresholds of variable importance in projection > 1, *P* < 0.05, 156 lipid species were identified as significantly altered between the DN and DN+DAPA groups (Fig. 1D). These included 48 neutral lipids, 66 phospholipids, and 42 sphingolipids (Fig. 1E). In detail, the differential lipids between the DN group and the DN+DAPA group were predominantly phosphatidylcholines (PC), accounting for 16.67% of the total, followed by ceramides (Cer) at 12.18%. Fatty acids (FA), hexosylceramides (HexCer), phosphatidylinositols (PI), triacylglycerols (TAG), and sulfoglycosphingolipids (SHexCer) also constituted notable proportions, accounting for 7.69%, 7.69%, 7.69%, 7.05%, and 5.77%, respectively (Fig. 1F). Additionally, significant differences were observed in various other lipid classes, including acylcarnitines (ACar), lysophosphatidylcholines (LPC), acylglucosyl diacylglycerols (AcylGlcADG), and diacylglyceryl trimethylhomoserines (DGTS). This overall distribution pattern suggests that dapagliflozin intervention exerts broad regulatory effects on the serum lipid profile in patients with diabetic nephropathy.

### The clinical predictive potential of dapagliflozin-responsive lipid species

**Fig. 2:**
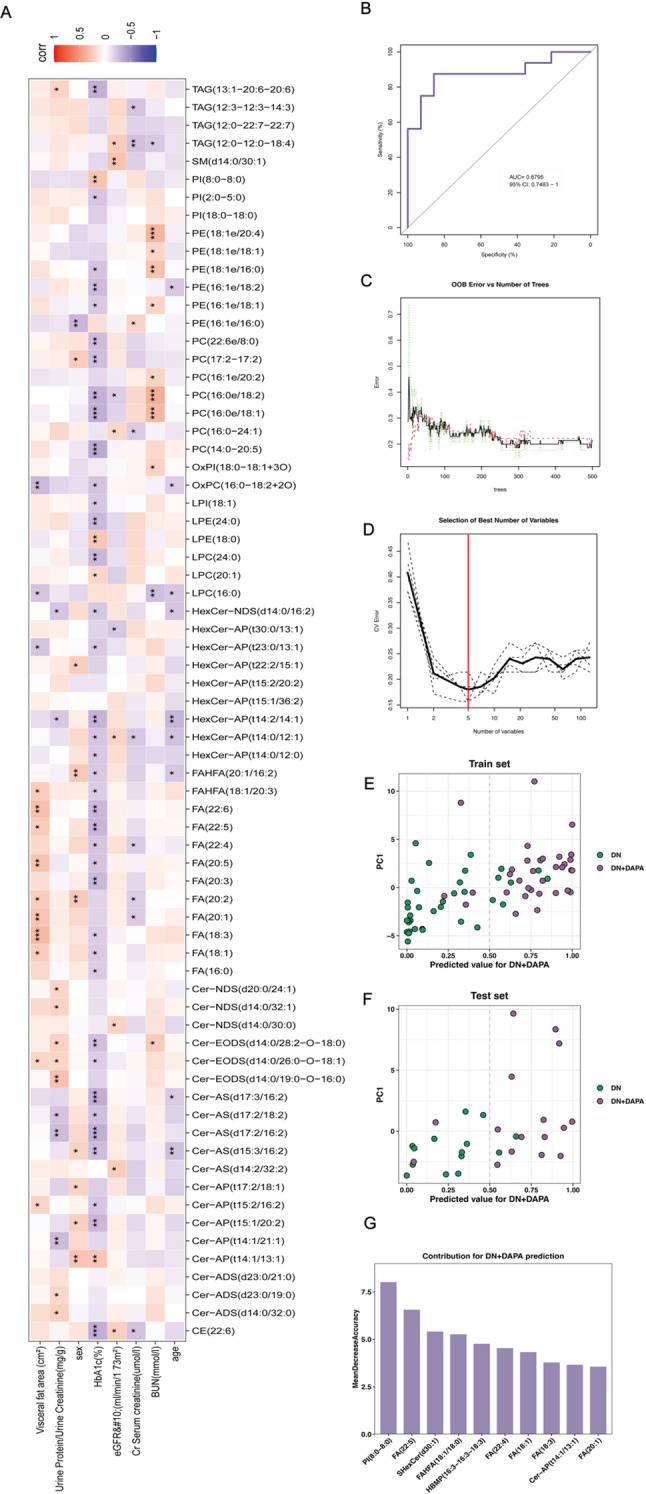
The predictive power of the difference lipid after dapagliflozin intervention for DN. **A**. Correlation between differential lipids and clinical renal injury indicators. **B-G**. Screening of differential lipids as potential predictors by random forest

The identification of dapagliflozin-altered lipids prompted an investigation into their correlations with renal function. We performed an integrated association analysis between differential lipid metabolites and key clinical indicators of DN, including eGFR, urinary albumin, serum creatinine, UACR, and HbA1c, comparing the DN+DAPA and DN groups. Hierarchical clustering heatmap analysis revealed distinct co-variation patterns between specific lipid species and clinical parameters (Fig. 2A). Notably, the sphingolipids SM(d14:0/30:1), HexCer-AP(t30:0/13:1), Cer-AS(d14:2/32:2), and Cer-NDS(d14:0/30:0) were positively associated with eGFR, suggesting that their increased levels may be linked to preserved renal function. In contrast, PC(16:0e/18:2) exhibited a negative correlation with eGFR. Several phospholipids, including PE(18:1e/20:4), PE(18:1e/16:0), PC(16:0e/18:2), and PC(16:0e/18:1), were positively correlated with BUN, whereas LPC(16:0) was negatively correlated. Additional associations were observed with glycemic control and renal injury markers. LPE(18:0), Cer-AP(t14:1/13:1), PI(8:0–8:0), and AcylGlcADG(12:0–16:3–16:3) correlated positively with HbA1c, while PI(2:0–22:2), ACar(15:0), and PC(16:0e/18:1) showed inverse relationships. PE(16:1e/16:0) was positively correlated with serum creatinine, whereas PC(14:0–22:3) was negatively correlated. Furthermore, the urinary albumin-to-creatinine ratio (UACR) displayed positive correlations with specific sphingolipid species. These results collectively indicate that dapagliflozin-induced lipid remodeling is closely linked to multiple clinical indicators of renal function, highlighting the potential of specific lipid species as biomarkers for DN progression and therapeutic response.

To further assess the predictive capacity of these differential lipid metabolites, a random forest algorithm was used to construct a classification model. The model demonstrated strong performance on the test set, achieving an area under the ROC curve of 0.8795 (Fig. 2B-D). Effective class separation was evident in the distribution of predicted probabilities, with samples from the DN and DN+DAPA groups clustering distinctly relative to the decision boundary (Fig. 2E-F). An optimal subset of variables was identified via ten-fold cross-validation, and variable importance analysis revealed that lipid species including FA (22:5), FAHFA(18:1/18:0), Cer-AP(t14:1/13:1), and PI(8:0–8:0) contributed most significantly to classification accuracy (Fig. 2G). These key lipids are consistent with prior correlation analyses and further highlight the predictive potential of specific lipid alterations for renal impairment.

Collectively, these findings further demonstrate that dapagliflozin ameliorates DN by modulating specific lipid metabolites. Moreover, machine learning analyses validate the clinical predictive value of these lipid alterations.

### Dapagliflozin ameliorates renal lipotoxicity and histological damage in *db/db* mice

To connect human serum lipidomic findings with target organ alterations and gain mechanistic insights, we established a diabetic mouse model treated with dapagliflozin. After 8 weeks of intervention, dapagliflozin-treated mice exhibited significant improvements in systemic metabolism, including reduced body weight, lower blood glucose levels, and decreased food intake, accompanied by an increase in rectal temperature, suggesting enhanced energy expenditure (Supplementary Fig. 1A-D).

Besides, dapagliflozin treatment significantly mitigated the elevation of serum creatinine levels and reduced the urinary albumin as well as urinary creatinine in *db/db* mice (Supplementary Fig. 1E-G). Histological assessment revealed severe renal injury in untreated *db/db* mice, characterized by tubular dilation, brush border loss, tissue vacuolization, and exacerbated fibrosis, as shown by H&E and Masson’s staining. PAS staining revealed significant mesangial matrix expansion and thickening of the glomerular basement membrane in *db/db* mice. Importantly, these structural lesions were markedly ameliorated in dapagliflozin-treated mice. Oil Red O staining further demonstrated substantial lipid droplet accumulation in the kidneys of *db/db* mice, a hallmark of renal lipotoxicity, which was effectively reduced following dapagliflozin intervention (Supplementary Fig. 1H). Collectively, these physiological and histopathological observations indicate that dapagliflozin not only exerts renoprotective effects but also directly mitigates renal lipid deposition and tissue damage in diabetic mice.

### Dapagliflozin treatment is associated with alterations in renal lipid distribution and metabolic pathway expression

**Fig. 3:**
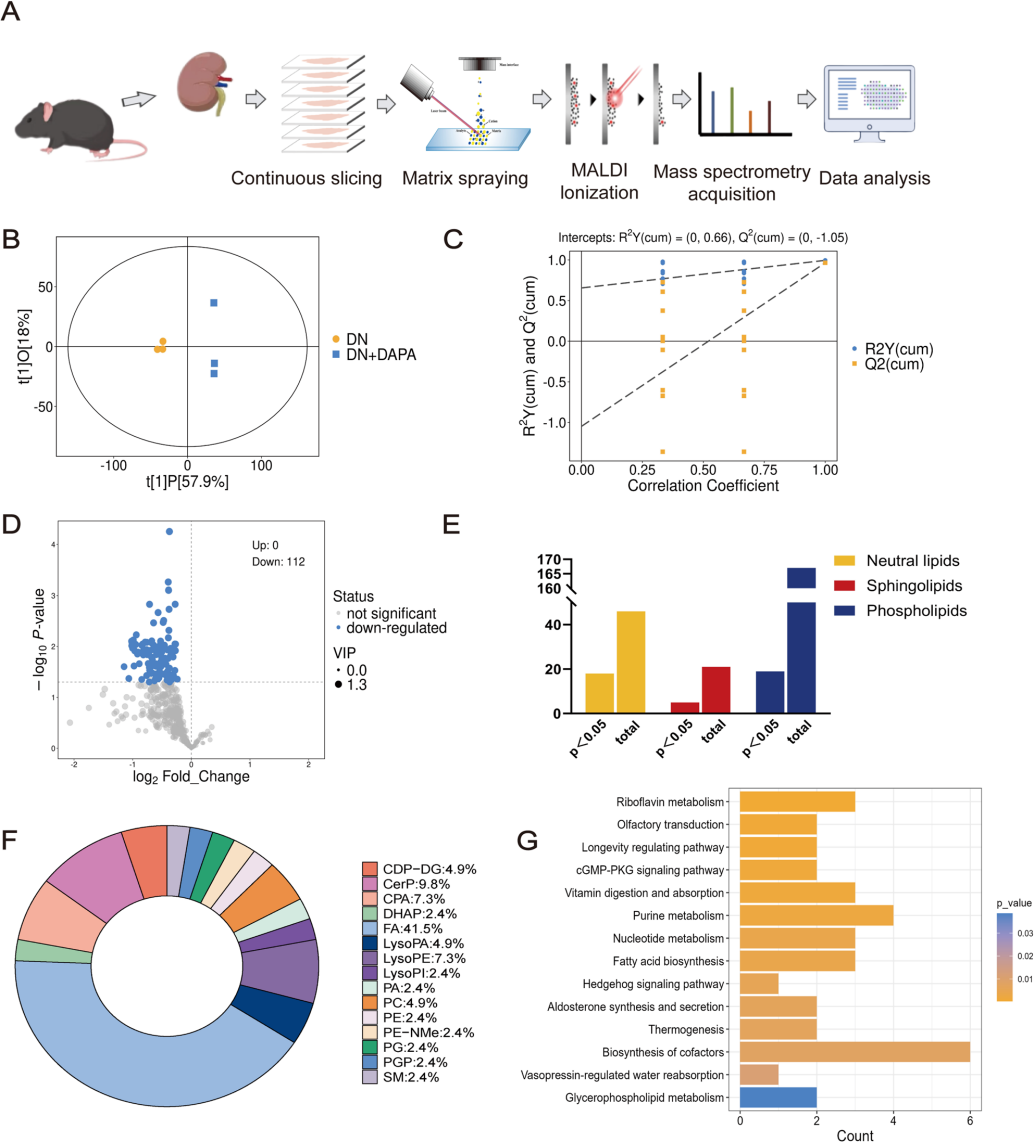
Dapagliflozin induces renal lipid remodeling and metabolic pathway alterations in diabetic mice. **A**. Schematic of the spatial metabolomics workflow using MALDI-MSI on kidney tissues. **B**. OPLS-DA score plots of DN group and DN+DAPA group based on MALDI-MSI data. **C**. Statistical validation of the OPLS-DA model through permutation testing. **D**. Volcano plots show the differential lipid alterations. **E**. The graph denotes the number of differential/non-differential abundances of lipid species. **F**. Pie charts show the differential abundance of lipid classes between two groups. **G**. Significantly enriched KEGG pathways, including fatty acid biosynthesis

Subsequently, high-resolution mass spectrometry imaging (MALDI-MSI) was employed to map the intrarenal distribution of discriminant metabolites on kidney tissues from *db/db* mice after 8 weeks of dapagliflozin treatment (Fig. 3A). OPLS-DA model revealed a clear separation between the DN group and the dapagliflozin-treated group (DN+DAPA), indicating a comprehensive reorganization of the renal lipid landscape in response to dapagliflozin (Fig. 3B). Permutation testing confirmed the absence of model overfitting and supported the robustness of the OPLS-DA model (Fig. 3C).

At 50 μm resolution, MALDI-MSI clearly delineated compartment-specific redistribution of key lipid metabolites. Volcano analyses identified 112 differentially abundant metabolites between the DN and DN+DAPA groups (Fig. 3D-E). In the spatial metabolomics analysis of mouse kidney tissue, the differential lipid molecules exhibited a distinct class distribution pattern. Fatty acids (FA) emerged as the most predominant class, accounting for 41.5% of the total and occupying an absolutely dominant position among all categories. Ceramide phosphates (CerP) constituted the second-largest class, representing 9.8%, a proportion significantly higher than that of most other lipid classes. Cyclic phosphatidic acids (CPA) and lysophosphatidylethanolamines (LysoPE) each accounted for 7.3%, also occupying notable proportions among the differential lipids. CDP-diacylglycerols (CDP-DG), lysophosphatidic acids (LysoPA), and phosphatidylcholines (PC) were each identified at a consistent proportion of 4.9%. The remaining lipid classes, including dihydroxyacetone phosphate (DHAP), lysophosphatidylinositol (LysoPI), phosphatidic acid (PA), phosphatidylethanolamine (PE), N-methylphosphatidylethanolamine (PE-NMe), phosphatidylglycerol (PG), phosphatidylglycerophosphate (PGP), and sphingomyelin (SM), were each detected at a proportion of 2.4%. Overall, the differential lipids were predominantly fatty acids, while also encompassing a diverse range of phospholipids, lysophospholipids, and sphingolipids. This distribution profile reflects the characteristics of renal lipid metabolism as elucidated by spatial metabolomics in mice (Fig. 3F).

Subsequent pathway enrichment analysis revealed that the glycerophospholipid metabolism pathway and the fatty acid biosynthesis pathway were significantly enriched. These findings are in line with the predominant distribution of phospholipids and fatty acids among the differential lipids, collectively suggesting dysregulated lipid metabolism in the mouse kidney at the spatial metabolomics level (Fig. 3G).

### Dapagliflozin promotes renal-systemic lipid redistribution through coordinated modulation of multiple pathways

**Fig. 4:**
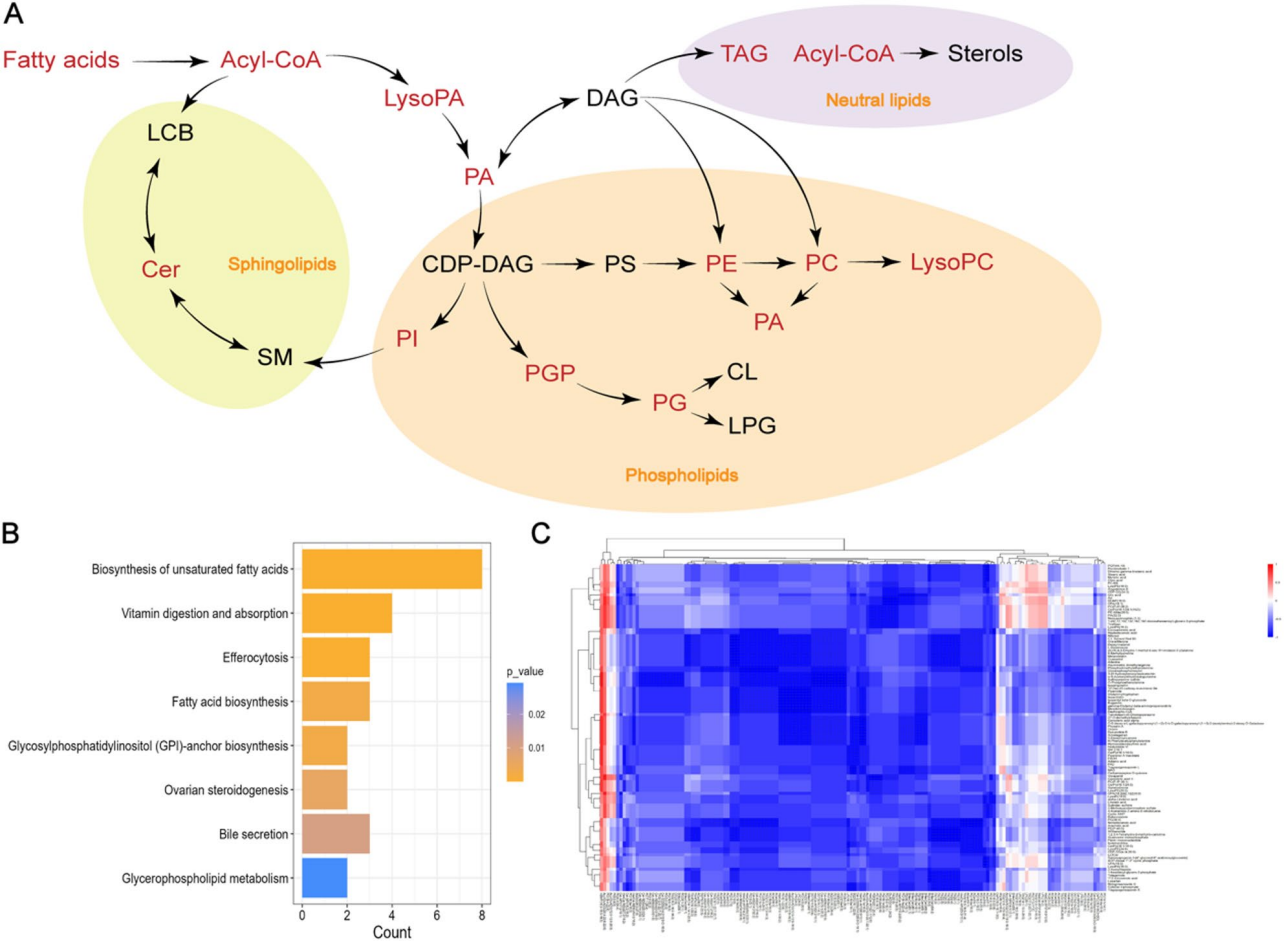
Multimodel joint analysis revealed the renal-circulatory lipid redistribution pathway. **A**. Simplified overview of lipid metabolism. **B**. Kegg pathway enrichment of joint analysis after dapagliflozin treatment. **C**. Spearman correlation analysis of lipid differences in human lipidomics and lipid differences in the spatial metabolomics of mouse kidneys

To integrate findings from human and murine studies, we combined serum lipidomics data with spatial metabolomics profiles. This cross-species analysis revealed associations between dapagliflozin treatment and alterations in lipid metabolic networks across systemic and renal compartments (Fig. 4A). KEGG pathway enrichment analysis identified key metabolic pathways modulated by dapagliflozin, including glycerophospholipid metabolism, sphingolipid metabolism, fatty acid biosynthesis and degradation, and fatty acid β-oxidation (Fig. 4B). Correlation analyses between circulating and renal lipids demonstrated significant inter-compartmental interactions. For instance, renal oleic and myristic acid levels correlated negatively with serum acylcarnitines ACar(17:1) and ACar(15:0), while renal stearic acid showed coordinated variations with serum long-chain fatty acids. These findings, together with the observed activation of saturated fatty acid β-oxidation, support enhanced fatty acid catabolism following treatment (Fig. 4C). This integration is intended to capture the systemic-to-local metabolic shifts, which is a common approach in multi-omics studies to identify robust biomarkers. Notably, associations between serum fatty acids in humans and renal phosphatidylglycerols in mice suggest reciprocal crosstalk between systemic and renal lipid metabolism. Furthermore, the observed negative correlations between human serum SM and murine renal Cer indicate that dapagliflozin may dynamically regulate lipid homeostasis by reducing renal lipid accumulation or enhancing lipid efflux, thereby coordinating metabolic balance across organ systems.

### Dapagliflozin may enhance lipid clearance by improving renal fatty acid oxidation

**Fig. 5:**
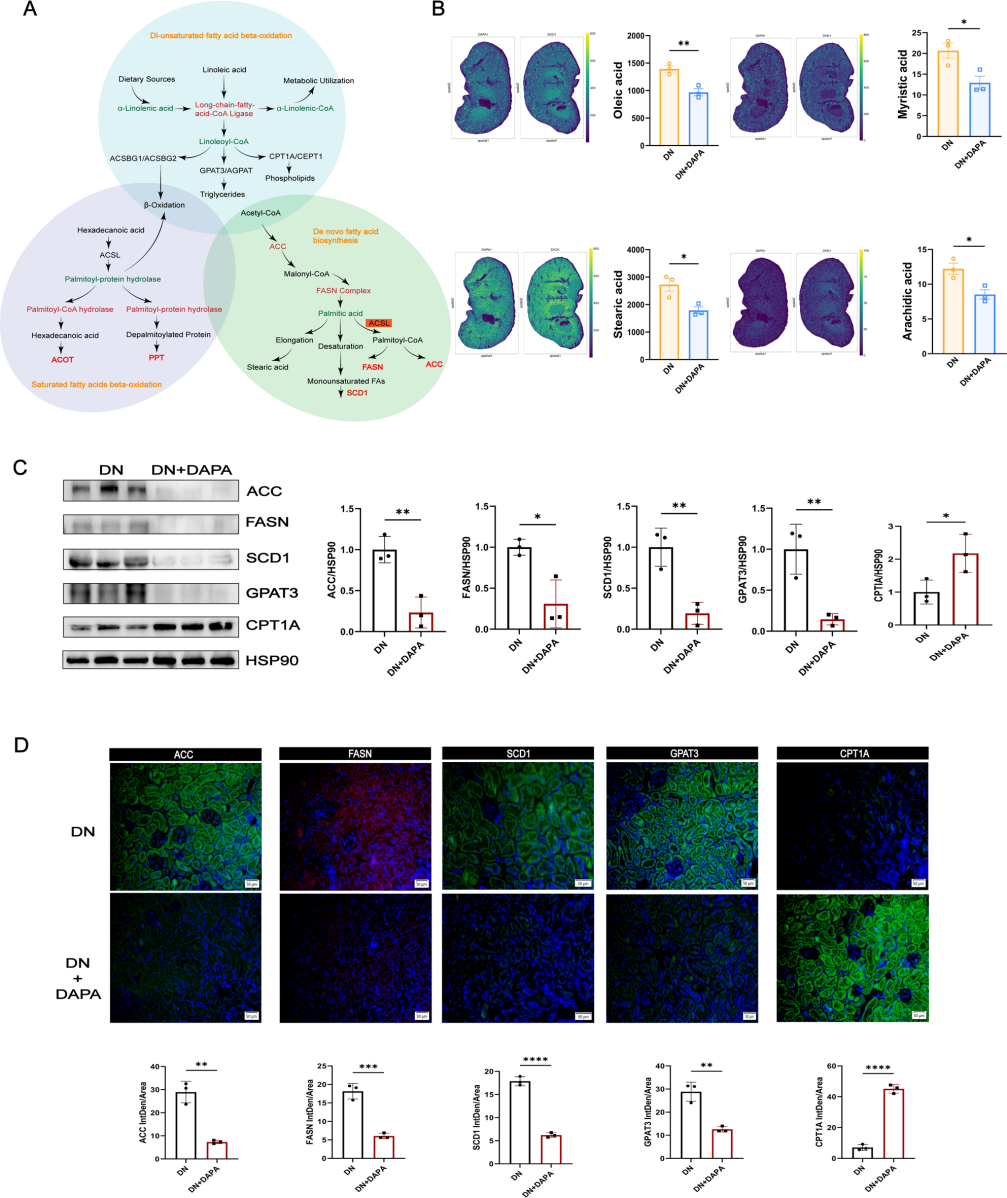
Dapagliflozin enhances renal fatty acid clearance by promoting β-oxidation and suppressing lipogenesis. **A**. Network analysis identifies fatty acid metabolism as a key target of dapagliflozin. **B**. Spatial metabolomics imaging reveals suppressed abnormal accumulation of toxic fatty acids in diabetic kidneys after DAPA treatment. **C**. Representative Western blots and quantification of ACC, FASN, SCD1, GPAT3, and CPT1A in renal tissues after DAPA treatment. **D**. Immunofluorescence staining and the quantifications of ACC, FASN, SCD1, GPAT3 and CPT1A in the kidney after DAPA treatment. *=*P* < 0.05, **=*P* < 0.01, ***=*P* < 0.001, ****=*P* < 0.0001

We first examined the effects of dapagliflozin on renal energy metabolism pathways (Fig. 5A). Spatial imaging revealed widespread abnormal accumulation of key fatty acids, including oleic acid, myristic acid, and stearic acid, in the renal cortex of diabetic mice, which was substantially reduced following dapagliflozin treatment (Fig. 5B). Changes in renal distribution of these saturated and monounsaturated fatty acids corresponded closely with circulating metabolite levels. The reduction in renal fatty acid accumulation aligns with increased β-oxidation activity, suggesting that dapagliflozin promotes lipid clearance by improving renal fatty acid oxidation capacity, thereby mitigating lipid-mediated toxicity.

We further examined the expression of key enzymes within the enriched pathways by Western blot and immunofluorescence. Dapagliflozin treatment was associated with altered expression of lipogenic enzymes, including reduced levels of ACC, FASN, GPAT3, and SCD1, alongside increased expression of CPT1A, a regulator of fatty acid β-oxidation (Fig. 5C-D). These enzymatic changes are consistent with a shift in lipid metabolism from *de novo* lipogenesis toward fatty acid catabolism; however, the functional consequences of these expression changes and their causal relationship to the observed lipidomic alterations require further investigation. This enzymatic shift provides a mechanistic explanation for the reduced lipid accumulation observed in our spatial metabolomics and Oil Red O staining analyses. Collectively, these results demonstrate that dapagliflozin effectively modulates renal fatty acid metabolic pathways.

### Dapagliflozin ameliorates circulatory metabolic abnormalities by modulating renal sphingolipid metabolism

**Fig. 6:**
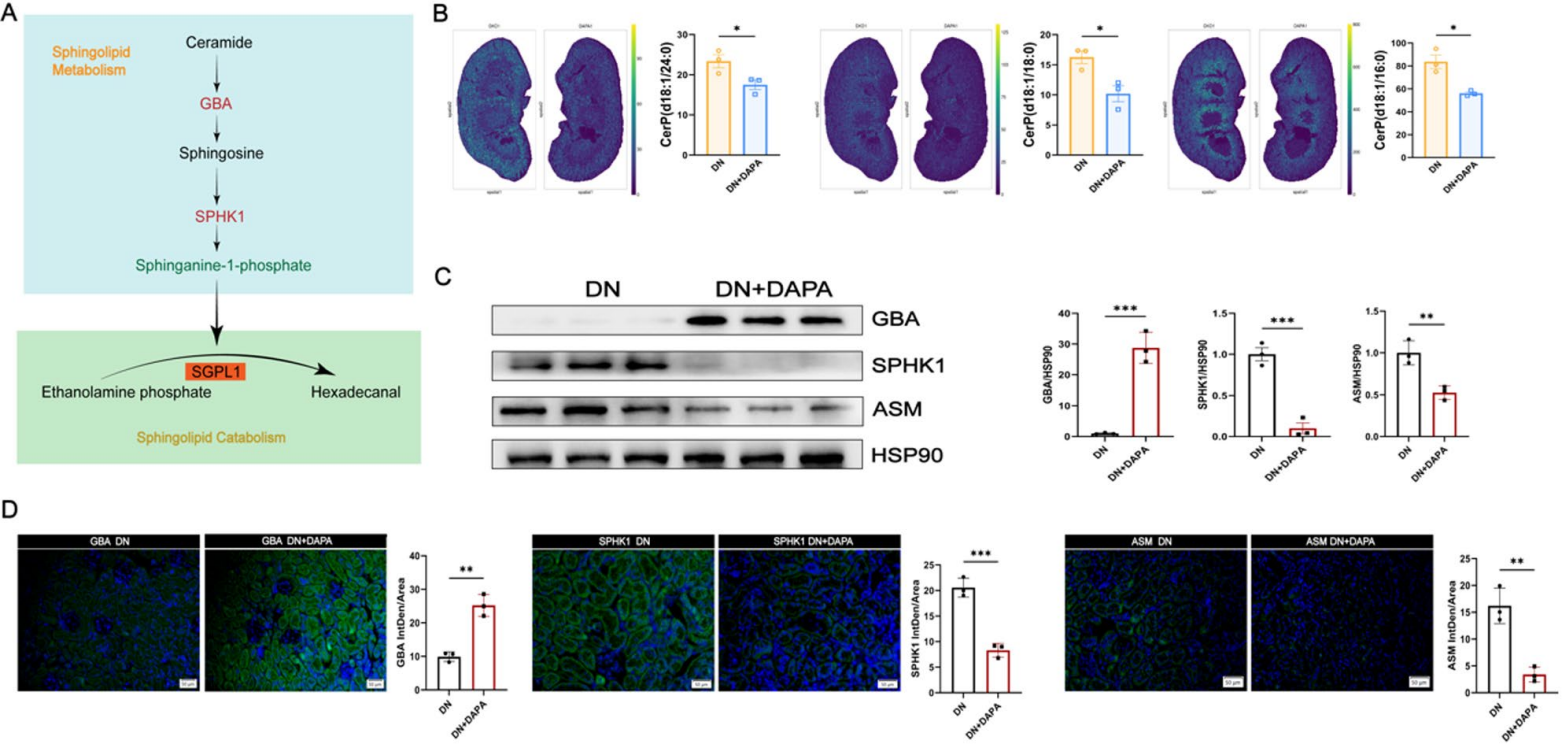
Dapagliflozin reprograms sphingolipid metabolism to alleviate renal lipotoxicity. **A**. Network analysis identifies sphingolipid metabolism as a key target of dapagliflozin. **B**. Spatial metabolomics imaging shows the decreased accumulation of ceramide-1-phosphates (CerPs) in diabetic kidneys after DAPA treatment. **C**. Representative Western blots and quantification in renal tissues. **D**. Representative immunofluorescence staining and the quantifications in the kidney after DAPA treatment. *=*P* < 0.05, **=*P* < 0.01, ***=*P* < 0.001

Building on dapagliflozin’s effects on energy metabolism, we next examined its impact on lipid signaling pathways. Network analysis identified sphingolipid metabolism as a key regulatory target of dapagliflozin (Fig. 6A). Spatial imaging revealed co-localized accumulation of Cer-1-phosphates, CerP(d18:1/16:0), CerP(d18:1/18:0), and CerP(d18:1/24:0), in both glomerular and tubulointerstitial regions of DN mice. This abnormal deposition was significantly ameliorated following dapagliflozin treatment (Fig. 6B). Notably, these local sphingolipid changes closely mirrored alterations observed in circulating metabolites. Cross-species correlation analysis further established functional networks linking renal CerP species with circulating sphingolipids, including HexCer-AP(t30:0/13:1) and SM(d14:0/30:1), highlighting coordinated regulation of sphingolipid metabolism across renal and systemic compartments.

Western blot and immunofluorescence analysis showed reduced expression of the sphingolipid-synthesizing enzyme SPHK1 and Acid sphingomyelinase (ASM, involved in ceramide production from sphingomyelin) and increased expression of GBA, an enzyme implicated in sphingolipid degradation (Fig. 6C-D). These expression changes are consistent with altered sphingolipid metabolism; however, whether these enzymatic alterations directly cause the observed changes in circulating sphingolipids remains to be determined.

### Dapagliflozin exerts renal and systemic remodeling of glycerophospholipid metabolism

**Fig. 7:**
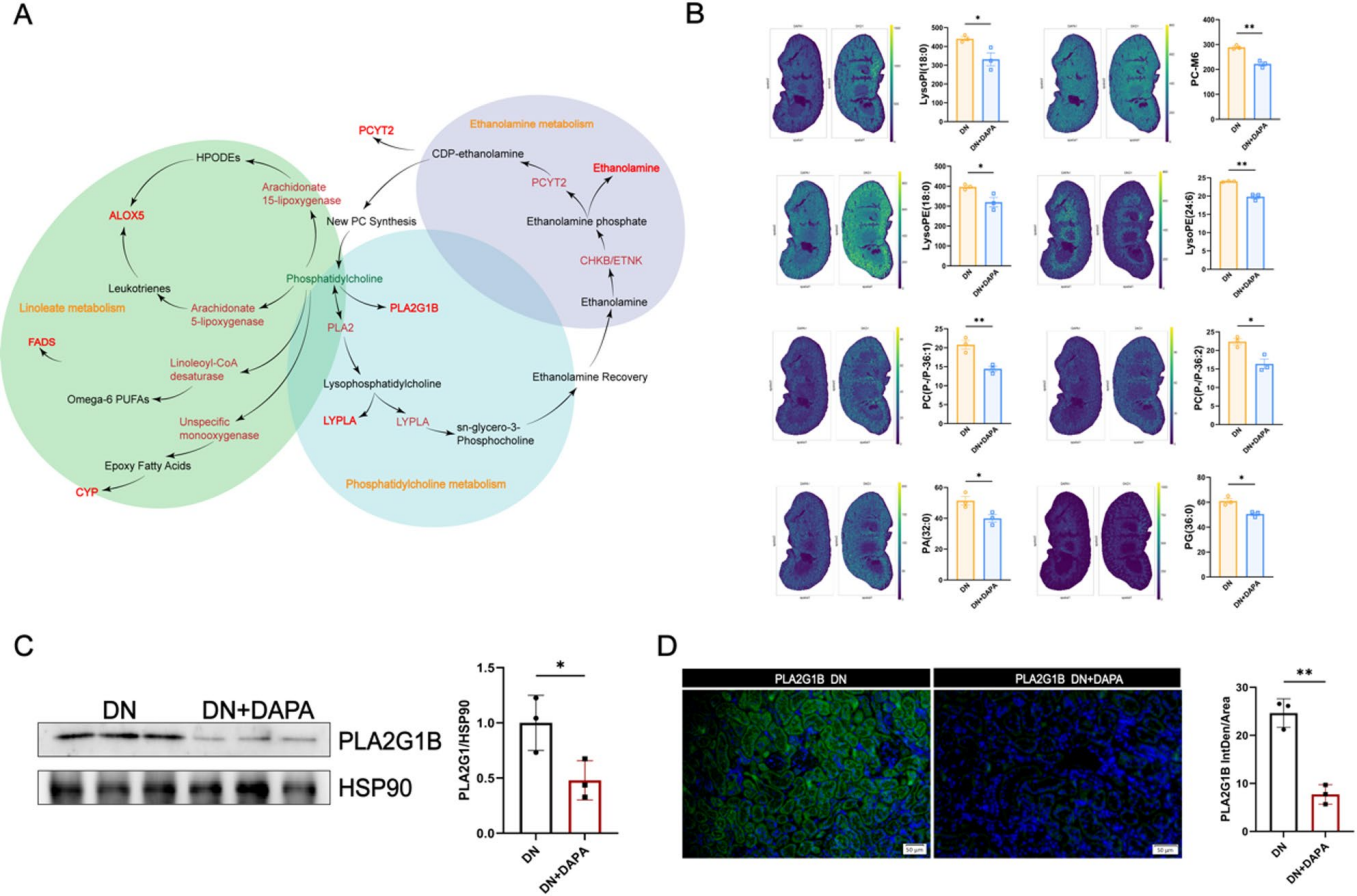
Dapagliflozin remodels glycerophospholipid metabolism in the diabetic kidney. **A**. Network analysis identifies glycerophospholipid metabolism as a central target. **B**. Spatial metabolomics imaging reveals reduced enrichment of lysophosphatidic acids (e.g., LysoPA(18:0), LysoPA(18:2)) and specific phosphatidylcholine (PC) subspecies in diabetic kidneys after DAPA treatment. **C**. Western blots of the expression of PLA2G1B and its quantification. **D**. Immunofluorescence staining and the quantification of PLA2G1B in the kidney after DAPA treatment. *=*P* < 0.05, **=*P* < 0.01

Network analysis identified glycerophospholipid metabolism as a key regulatory target, serving as a primary structural component of cellular membranes. Our findings highlight glycerophospholipid metabolism as a central pathway through which dapagliflozin exerts renoprotective effects (Fig. 7A). Spatial imaging revealed marked enrichment of LysoPA(18:0) and LysoPA(18:2) in the renal cortex of DN mice, which was effectively attenuated following dapagliflozin treatment. These results indicate that LysoPA metabolism links local renal lipid dynamics to systemic circulation. Moreover, the observed spatial distribution changes in the kidney were consistent with serum lipidomic profiles. Specifically, renal LysoPA(18:2) levels correlated negatively with circulating PCs(15:0–16:1) and PC(17:0–20:5), while renal LysoPA(18:0) showed a negative correlation with the serum fatty acid ACar(15:0).

PC serves as a central component of glycerophospholipids, and specific PC subtypes, including PC-M6, PC(P-P-36:1), and PC(P-P-36:2), exhibited distinct spatial distribution patterns in diabetic kidneys. Dapagliflozin treatment effectively remodeled these patterns, normalizing their intrarenal localization. Correlation analyses revealed that these renal PC subspecies were closely associated with multiple circulating PC species, highlighting the integration of tissue and systemic lipid pools. These findings indicate that dapagliflozin facilitates coordinated lipid redistribution between renal and circulatory compartments through modulation of PC metabolism.

Further investigation confirmed that PLA2G1B was significantly downregulated. This enzyme plays a key role in the generation of lysophospholipids (Fig. 7B-D), and its suppression provides a mechanistic explanation for the favorable remodeling of renal phospholipids observed following dapagliflozin treatment.

These findings suggest an association between glycerophospholipid metabolism alterations and changes in both renal lipid distribution and systemic lipid profiles. In summary, dapagliflozin treatment was associated with multi-level alterations in glycerophospholipid metabolism markers, spanning pro-inflammatory LysoPA to structural PCs. While these associations are consistent with a coordinated regulatory mechanism, direct functional studies are required to establish causality and to confirm that these lipid metabolic changes are responsible for the observed renoprotective effects.

## Discussion

This study reveals associations between dapagliflozin treatment and alterations in lipid metabolism across both systemic and renal compartments in DN. By integrating clinical lipidomics with high-resolution spatial metabolomics of renal tissues, we identify coordinated changes in circulating lipid profiles and intrarenal lipid distribution that are associated with improved renal function. These effects are mediated, at least in part, via transcriptional regulation of key lipid-metabolizing enzymes, culminating in the restoration of systemic metabolic homeostasis. Collectively, our findings provide novel insights into the potential lipid-mediated mechanisms underlying dapagliflozin’s multi-organ protective effects.

Our clinical and lipidomic analyses show that dapagliflozin treatment is associated with significant alterations in lipid profiles in patients with DN. Treatment with dapagliflozin was accompanied by reorganization of the serum lipidome, characterized by increased PCs alongside reductions in LPC and triglycerides. These lipid alterations are consistent with potential mechanisms including enhanced renal lipid efflux and suppression of lysophospholipid-mediated inflammatory pathways, however, the specific mechanisms driving these changes and their direct contribution to the observed renal benefits remain to be elucidated. Our findings align with previous reports on the lipid-modifying effects of SGLT2 inhibitors [33, 34]. The observed decrease in neutral lipids, particularly TAGs, aligns with dapagliflozin’s known role in promoting renal lipolysis and fatty acid β-oxidation [35]. Moreover, modulation of sphingolipids and anti-inflammatory lipids may contribute centrally to dapagliflozin’s renoprotective effects. Renal Cer accumulation has been reported to exacerbate insulin resistance and renal injury [36–38], and the elevated levels of circulating sphingolipids observed here suggest that dapagliflozin may facilitate Cer clearance from the kidney. Upregulation of anti-inflammatory FAHFAs further highlights the drug’s systemic lipid-modulating effects [39]. Importantly, machine learning analyses identified specific lipid species, including SM(d14:0/30:1) and PC(16:0e/18:2), that were strongly correlated with improved renal function, as measured by eGFR and BUN. Collectively, these findings indicate that dapagliflozin-mediated suppression of pro-inflammatory and pro-fibrotic lipids, together with enhancement of renoprotective species, underlies its clinical efficacy and highlights the potential of these lipids as biomarkers for DN progression and therapeutic response.

Beyond its systemic effects, our spatial metabolomics data visualize dapagliflozin-associated alterations in lipid distribution within the diabetic kidney. Previous studies have established that lipids associated with inflammation and oxidative stress accumulate in renal tissue [40, 41]. We observed a marked reduction in the renal accumulation of toxic lipid species, including saturated fatty acids (e.g., stearic acid) and monounsaturated fatty acids (e.g., oleic acid), which are known to activate injurious pathways such as those mediated by PPARs [42, 43]. This attenuation of renal lipotoxicity was accompanied by changes in PC subspecies distribution and a pronounced decrease in precursors involved in cardiolipin biosynthesis (e.g., LysoPA(18:2), PA(32:0)). Given the established role of dysregulated cardiolipin remodeling in mitochondrial dysfunction and DN progression [44–46], these changes suggest a novel mechanism through which dapagliflozin preserves mitochondrial integrity in the kidney. Concurrnt reductions in CerPs [47–51] and lysophospholipids (LysoPE, LysoPI) [52, 53] further support dapagliflozin’s role in mitigating renal inflammatory signaling and cellular toxicity. Together, these findings indicate that the renoprotective effects of dapagliflozin are closely linked to its capacity to remodel the intrarenal lipid environment, thereby alleviating localized, lipid-mediated damage as revealed by spatially resolved metabolomic profiling.

Integration of systemic and renal lipidomic datasets revealed associations consistent with a coordinated model of dapagliflozin-related metabolic alterations. Our analyses indicate correlations suggesting that dapagliflozin coordinately regulates a network of metabolic pathways, simultaneously enhancing catabolic processes such as fatty acid β-oxidation while suppressing anabolic pathways, including *de novo* lipogenesis and glycerophospholipid/sphingolipid synthesis. This metabolic shift extends beyond the kidney, as reflected by reciprocal changes between renal and circulating lipid pools. For example, reductions in renal CerPs correlated with specific alterations in circulating sphingolipids, suggesting integrated modulation of inflammatory and fibrotic pathways across organ systems [54–56]. This system-level metabolic reprogramming likely contributes to the improvements in insulin sensitivity and the reduction of lipotoxicity observed in our study and may also help explain the cardioprotective effects associated with dapagliflozin [57, 58]. The decrease in renal oleic acid accumulation, for instance, may influence systemic lipid profiles in a manner that mitigates cardiac lipotoxicity, consistent with epidemiological studies linking oleic acid levels to cardiovascular risk [59, 60]. Collectively, these observations align with the emerging concept that SGLT2 inhibitors induce a systemic “fasting-like” metabolic state, characterized by a shift in substrate utilization from carbohydrates to lipids, providing a unifying physiological rationale for their multi-organ benefits [17, 61, 62].

At the molecular level, our experimental data identify key enzymatic drivers underlying dapagliflozin-induced metabolic reprogramming. Dapagliflozin transcriptionally modulated a series of enzymes, suppressing key lipogenic enzymes, including ACC, FASN, GPAT3, and SCD1, while upregulating CPT1A, a critical regulator of fatty acid β-oxidation [63, 64]. Within the sphingolipid pathway, it restored homeostasis by upregulating the degradative enzyme GBA and downregulating the synthetic enzyme SPHK1, thereby reducing levels of the pro-fibrotic mediator S1P [65–67]. Additionally, downregulation of PLA2G1B provides a mechanistic explanation for the observed decrease in pro-inflammatory lysophospholipids [68–72]. This coordinated regulation across multiple enzymatic pathways suggests that dapagliflozin acts via a central regulatory mechanism. Although our study did not directly measure AMPK activity, the observed pattern, suppression of ATP-consuming biosynthetic processes alongside stimulation of ATP-generating oxidative pathways, strongly implicates AMP-activated protein kinase (AMPK) as a central orchestrator [73]. We propose a speculative model in which dapagliflozin-mediated inhibition of glucose reabsorption in the proximal tubule may induce a mild intracellular energy deficit, potentially activating AMPK. Activated AMPK could then trigger transcriptional reprogramming of lipid-metabolic enzymes, contributing to the metabolic alterations observed in both our lipidomic and spatial metabolomic analyses.

### Limitations

Several limitations of this study should be acknowledged. The absence of a placebo or standard-treatment control group in our clinical cohort represents a limitation in establishing direct causality between dapagliflozin and the observed lipidomic changes. Although we demonstrate robust associations between dapagliflozin treatment, lipidomic remodeling, and improved renal outcomes, the precise molecular sensor that links SGLT2 inhibition to the observed transcriptional changes in lipid-metabolizing enzymes remains unidentified. Functional validation through targeted manipulation of these pathways (e.g., selective enzyme knockout, lipid supplementation/depletion) would be required to establish the causal sequence of events and to confirm which of these pathway alterations are necessary and sufficient for mediating dapagliflozin’s renoprotective effects. Finally, while our data suggest involvement of AMPK, they remain largely hypothetical and require direct experimental validation, including measurement of renal AMPK phosphorylation and activity following dapagliflozin treatment; genetic or pharmacological AMPK manipulation studies to establish its necessity for dapagliflozin’s effects in future experimental studies.

## Conclusion

Using an integrative, cross-species approach that combines clinical lipidomics, high-resolution spatial metabolomics, and molecular validation, this study reveals multiple associations between dapagliflozin treatment and alterations in lipid metabolism. We show that dapagliflozin changes both systemic circulation and the intrarenal lipid environment to alleviate lipotoxicity, enhance mitochondrial function, and restore metabolic homeostasis. Collectively, these findings suggest that lipid metabolism represents a promising therapeutic target in DN and provide a foundation for future mechanistic studies to elucidate the precise role of specific lipid species and metabolic enzymes in mediating the renoprotective effects of SGLT2 inhibitors.

## Materials and methods

### Study design and grouping

We enrolled diabetic kidney injury patients treated with dapagliflozin at Huai’an First People’s Hospital between 2019 and 2022. A total of 51 patients (including 37 male patients and 14 female patients) were enrolled. To minimize baseline confounding, strict inclusion criteria were applied: (1) age between 25 and 75 years; (2) diagnosed of T2DM according to the World Health Organization (WHO) criteria; (3) glycated hemoglobin (HbA1c) levels between 6.0% and 13.0%; (4) presence of albuminuria, defined as a urinary albumin-to-creatinine ratio (UACR) of 30–2500 mg/g; (5) estimated glomerular filtration rate (eGFR) ≥ 45 mL/min/1.73 m². T2DM was diagnosed according to the World Health Organization (WHO) criteria, defined as a fasting plasma glucose (FPG) ≥ 7.0 mmol/L, a 2-hour post-load glucose ≥ 11.1 mmol/L during an oral glucose tolerance test (OGTT), or a glycated hemoglobin (HbA1c) ≥ 6.5%. Participants were excluded if they had pre-existing type 1 diabetes mellitus; secondary renal diseases other than diabetic kidney disease; active urinary tract infection; severe cardiovascular or cerebrovascular diseases; severe hepatic or renal impairment, including a history of renal dialysis or transplantation; pregnancy, lactation, or positive pregnancy test at screening; women of childbearing potential not using effective contraception (e.g., sterilization, intrauterine devices, oral contraceptives, or barrier methods); use of SGLT2 inhibitors, pioglitazone, GLP-1 receptor agonists, or DPP-4 inhibitors within 3 months prior to enrollment; systemic corticosteroid therapy or use of any investigational drugs within 2 months prior to screening.

This study employed a longitudinal design to evaluate the effects of dapagliflozin treatment. Participants were categorized into two groups based on the duration of treatment: 0-week group (Baseline): Patients prior to the initiation of dapagliflozin therapy. 12-week group: Patients after 12 weeks of continuous dapagliflozin treatment.

Ethical approval was obtained from the institutional review board (YX-P-2020-041-01), and written informed consent was provided by all participants.

Demographic and clinical data included sex, age, diabetes duration, BMI, smoking history, alcohol use, and hypertension history. Fasting venous blood was collected after 8–12 h of overnight fasting. Fasting blood glucose, total cholesterol, LDL-cholesterol, HDL-cholesterol, and triglycerides were analyzed by the hospital’s clinical laboratory. Visceral fat area and body fat percentage were measured by the radiology department.

### Sample collection and preparation

Blood samples were collected from 51 patients with diabetic kidney injury who were treated with dapagliflozin at baseline (0-week, pre-dapagliflozin) and after 12 weeks of treatment. In total, 102 serum samples (51 pairs) were analyzed. Statistical analyses, including paired t-tests or Wilcoxon signed-rank tests, were performed to account for the paired nature of the data. After collection, samples were immediately placed on ice, centrifuged at 3000 rpm for 10 min to obtain supernatants, and the supernatants were stored at − 80 °C.

### LC-MS/MS analysis and data processing

Untargeted lipidomics was performed using an ultra-high-performance liquid chromatography (UHPLC) system (Vanquish, Thermo Fisher Scientific) coupled with an Orbitrap Exploris 120 mass spectrometer (Thermo Fisher Scientific). Chromatographic separation was achieved on a Phenomenex Kinetex C18 column (2.1 mm × 100 mm, 2.6 μm). The mobile phase consisted of (A) H_2_O/ACN (6:4, v/v) and (B) IPA/ACN (9:1, v/v), both containing 10 mM ammonium formate.

Mass spectrometry data were acquired in data-dependent acquisition (DDA) mode. The full MS resolution was set at 60,000, and MS/MS resolution at 15,000. ESI source parameters were as follows: sheath gas flow rate 30 Arb, auxiliary gas flow rate 10 Arb, capillary temperature 320 °C, and spray voltage 3.8 kV (positive) or -3.4 kV (negative). Collision energies were set at 15, 30, and 45 eV. Raw data were converted to mzXML format using ProteoWizard (msconvert). Peak detection, alignment, and integration were processed using the CentWave algorithm in XCMS (minfrac = 0.5, cutoff = 0.3). Lipid identification was performed by spectral matching against the LipidBlast library.

Data preprocessing involved filtering features with > 50% missing values in any group or > 50% across all groups, followed by missing value imputation (minimum value × random number between 0.1 and 0.5) and normalization using internal standards. Log2-transformed data were imported into SIMCA 16.0.2 for multivariate analysis, including principal component analysis (PCA) and orthogonal partial least squares-discriminant analysis (OPLS-DA). Differentially expressed metabolites were identified using variable importance in projection (VIP) > 1 and Student’s t-test *P* < 0.05. Pathway analysis was performed via the Kyoto Encyclopedia of Genes and Genomes (KEGG) database.

### Correlation and random forest analysis

Spearman correlation analysis was performed to assess associations between the differential lipid metabolites and clinical parameters of renal function (including eGFR, BUN, serum creatinine, UACR, and HbA1c). Results were visualized as a hierarchical clustering heatmap. For predictive modeling, a random forest algorithm was implemented using the significant lipid species. The dataset was split into training (70%) and test (30%) sets. The model was built with 500 trees (ntree), and the optimal number of variables at each split was determined through ten-fold cross-validation. Model performance was evaluated on the test set by ROC analysis, and variable importance was assessed based on the MeanDecreaseAccuracy metric.

Regarding random forest validation, the samples were divided into a training set and a test set at a ratio of 7:3. To select the optimal feature subset from the high‑dimensional feature space, a recursive feature elimination with cross‑validation (RFECV) approach based on random forest was applied to the training set. Specifically, a ten‑fold stratified cross‑validation framework was used as the core validation scheme. Within each fold, a random forest model was built on the training split, and variable importance was computed. Feature counts were gradually reduced on a logarithmic scale (step = 0.7). For each feature subset, a model was trained, and the classification error was evaluated on the corresponding validation split. This procedure was repeated five times. The mean and standard deviation of the cross‑validated errors across different feature subset sizes were compared, and the feature count yielding the smallest cross‑validation error was selected as the optimal feature set. A final random forest model was then constructed using the selected features. Classification results for both the training and test sets were output, and model performance was assessed using receiver operating characteristic (ROC) curves and the area under the curve (AUC).

### Sample preparation for spatial metabolomics

Male *db/db* mice (7 weeks old) were obtained from GemPharmatech. After acclimatized for 1 week, *db/db* mice were then randomized into two groups. Experimental group received daily oral gavage of dapagliflozin (1 mg/kg) while control group received the same amount of PBS. After 8 weeks of intervention, left kidneys of mice were harvested, rinsed in ice-cold PBS, flash-frozen in CMC. Specimens were stored at -80 °C until cryosectioning and MALDI-TOF MS imaging (50 μm resolution, m/z 50-1200) at BioTree Company.

### Matrix-assisted laser desorption/ionization–mass spectrometry imaging and data preprocessing

Matrix-coated slides were loaded onto the target plate. Instrument calibration included target height profiling, laser focus adjustment, and mass axis calibration (using sodium formate clusters). Tissue regions were defined in FlexImaging 5.0 (Bruker Daltonics), followed by negative-ion mode imaging at 50-µm spatial resolution (m/z 50-1200). Raw data were imported into SCiLS™ Lab 2024 (Bruker) for baseline subtraction, peak alignment, spectral smoothing, and root mean square (RMS) normalization, generating spatially resolved intensity maps for m/z features.

### H&E staining

Kidney tissues fixed in 10% neutral-buffered formalin (24 h, 4℃) were processed through graded ethanol series, embedded in paraffin, and sectioned at 4-µm thickness. Sections were deparaffinized in xylene and rehydrated through descending ethanol concentrations. Following Sevier-Munger’s modified protocol, nuclei were stained with Mayer’s hematoxylin (8 min), differentiated in 1% acid ethanol (5 s), and blued in 0.2% ammonia water. Cytoplasmic counterstaining was performed with eosin Y (0.5%, 1 min). Dehydrated sections were cleared in xylene and mounted with neutral balsam.

### Masson staining

Kidney Sect.  (4-µm) were deparaffinized and rehydrated through xylene-ethanol series. Following Bouin’s fluid fixation (37 °C, 1 h) and Weigert’s iron hematoxylin nuclear staining (10 min), sections were sequentially treated with Biebrich scarlet-acid fuchsin (5 min), phosphomolybdic-phosphotungstic acid solution (10 min), and aniline blue (5 min) for differential staining of cytoplasm, connective tissue, and collagen. After 1% acetic acid differentiation (1 min), sections were dehydrated, cleared in xylene, and mounted with resinous medium.

### PAS staining

Fixed in 4% paraformaldehyde, paraffin‑embedded kidney tissues were sliced into 5‑µm sections. After dewaxing and rehydration, slides were treated with 0.5% periodic acid (10 min, room temperature), briefly rinsed with distilled water, and then incubated with Schiff’s reagent (15 min, dark). Following a 5‑min tap‑water wash, sections were counterstained with Mayer’s hematoxylin (3 min), dehydrated through an ethanol gradient, cleared in xylene, and mounted with neutral resin. Under this protocol, PAS‑positive structures (e.g., glycogen, basement membranes) appeared magenta, and nuclei appeared blue.

### Oil Red O staining

Cryosections of kidney tissue (6-µm thickness) were fixed in formalin and stained with a filtered Oil Red O working solution to visualize neutral lipids. Sections were then counterstained with hematoxylin to identify cellular nuclei. Lipid droplets were observed as red deposits under a light microscope.

### Western blotting

Renal tissues from *db/db* mice were homogenized in RIPA lysis buffer containing protease and phosphatase inhibitors. Protein concentrations were determined using a BCA assay. Equal amounts of protein were separated by SDS-PAGE and transferred to PVDF membranes. After blocking with 5% non-fat milk, membranes were incubated overnight at 4 °C with primary antibodies against ACC, FASN, SCD1, GPAT3, CPT1A, GBA, SPHK1, ASM and PLA2G1B (Proteintech, Wuhan, China). Following incubation with HRP-conjugated secondary antibodies, protein bands were visualized using enhanced chemiluminescence and quantified by densitometry.

### Immunofluorescence staining

Paraffin-embedded kidney sections were deparaffinized in xylene and rehydrated through a graded ethanol series. After antigen retrieval, sections were blocked with 5% BSA and then incubated overnight at 4°C with primary antibodiesagainst ACC, FASN, SCD1, GPAT3, CPT1A, GBA, SPHK1, ASM and PLA2G1b (all from Proteintech, Wuhan, China). After washing, slides were incubated with fluorophore-conjugated secondary antibodies and counterstained with DAPI. Images were captured using a fluorescence microscope.

### Statistical analysis

Statistical analyses were performed using GraphPad Prism and R software. Data are presented as mean ± SEM. Differences between two groups were assessed using unpaired or paired Student’s t-tests, as appropriate. A p-value of less than 0.05 was considered statistically significant.

## Supplementary Information

Below is the link to the electronic supplementary material.

Supplementary Material 1

Supplementary Material 2

Supplementary Material 3

Supplementary Material 4

## Data Availability

The data that support the findings of this study are available from the corresponding author upon reasonable request. Clinical data are not publicly available to protect patient privacy but may be made available to qualified researchers subject to a data sharing agreement and with appropriate ethical approvals.
