# Supplementary material for: Dapagliflozin induces renal lipidomic remodeling and systemic metabolic improvement
Source: Biol Direct. 2026 Apr 17;21:50. doi: 10.1186/s13062-026-00800-9 (PMC13094247; doi:10.1186/s13062-026-00800-9)
Supplement: Supplementary file 1 — Supplementary Material 1 [file 13062_2026_800_MOESM1_ESM.docx]

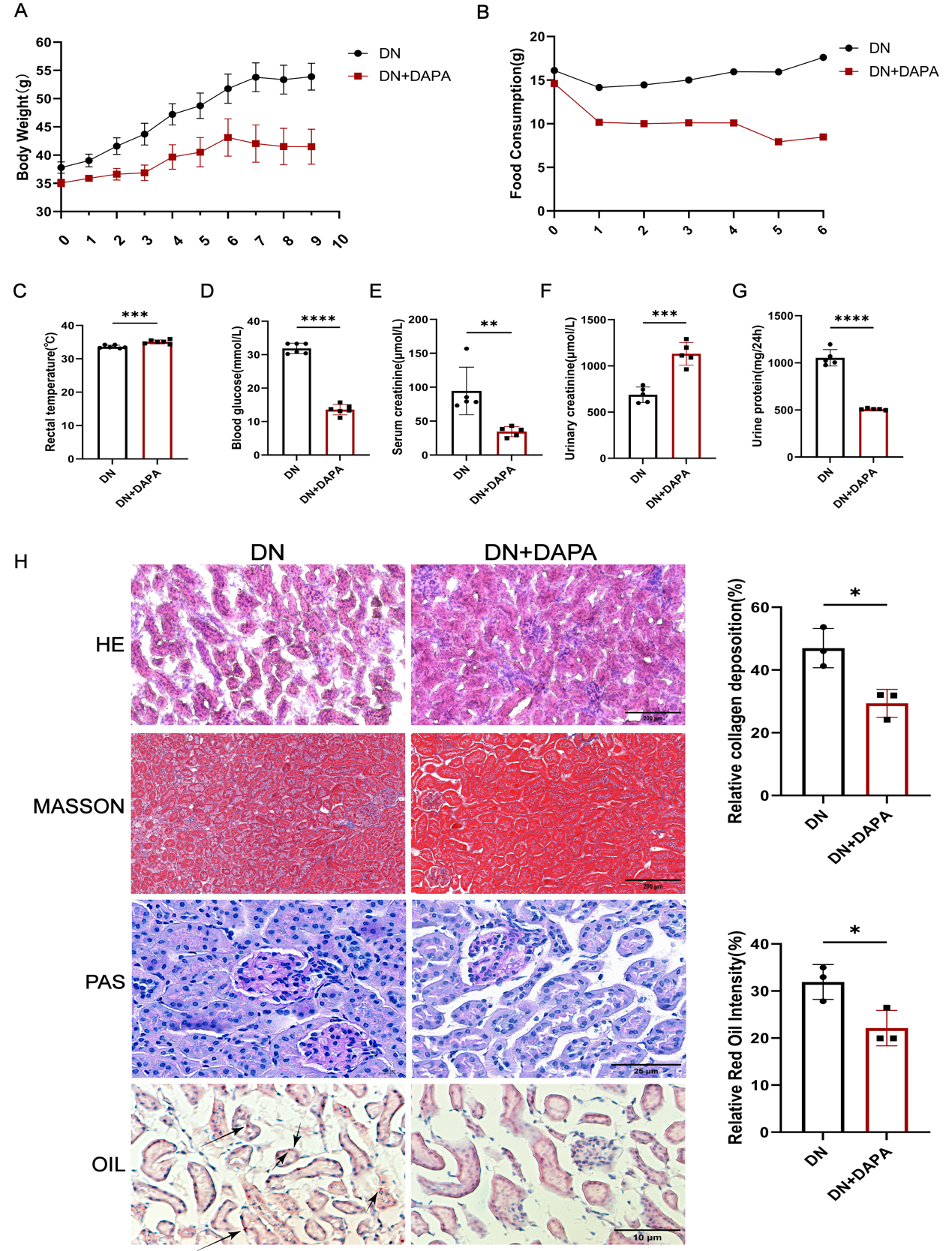


Supplementary 1. After dapagliflozin intervention, the pathological injury of db/db mice changed and renal injury was reduced.

A-D. Changes in body weight, food intake, blood glucose and rectal temperature of mice after dapagliflozin intervention.

E-G. Serum creatinine, urinary protein and urinary creatinine levels in *db/db* mice after dapagliflozin treatment.

H. H&E staining, Masson staining, PAS staining, and Oil Red O staining of mouse kidney sections and the quantitative statistics of H&E staining and Oil Red O staining.

*=P<0.05, **=P<0.01, ***=P<0.001, ****=P<0.0001.
