# Supplementary material for: Dapagliflozin induces renal lipidomic remodeling and systemic metabolic improvement
Source: Biol Direct. 2026 Apr 17;21:50. doi: 10.1186/s13062-026-00800-9 (PMC13094247; doi:10.1186/s13062-026-00800-9)
Supplement: Supplementary file 3 — Supplementary Material 3 [file 13062_2026_800_MOESM3_ESM.docx]

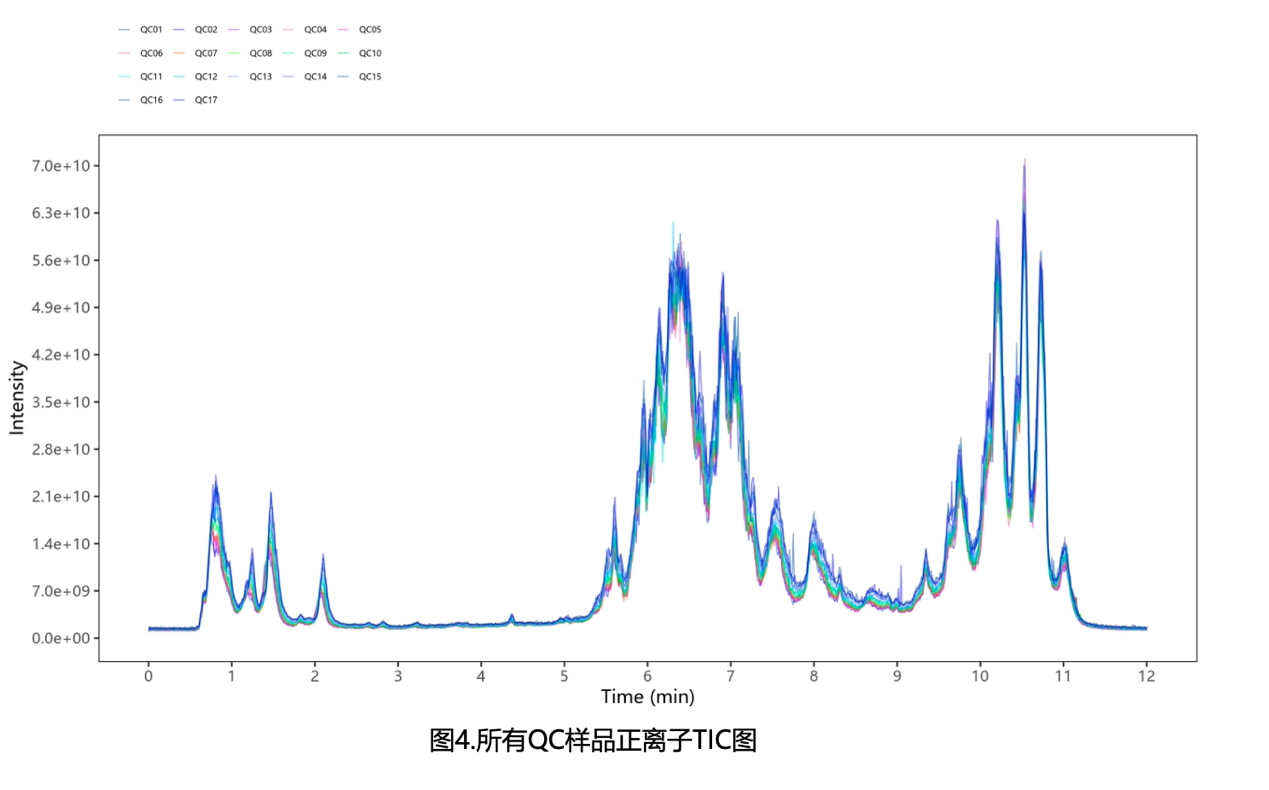


**Total ion current (TIC) chromatograms of all QC samples in positive ion mode.**


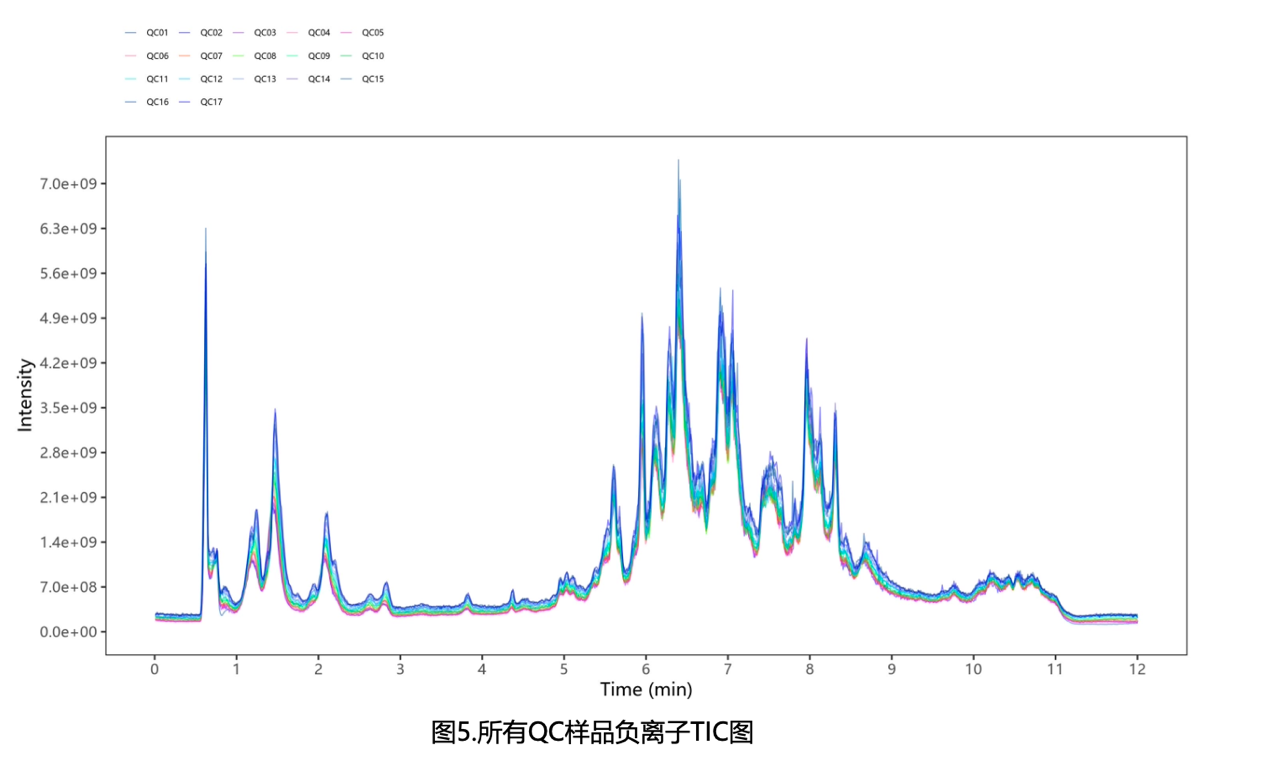


**Total ion current (TIC) chromatograms of all QC samples in negative ion mode.**


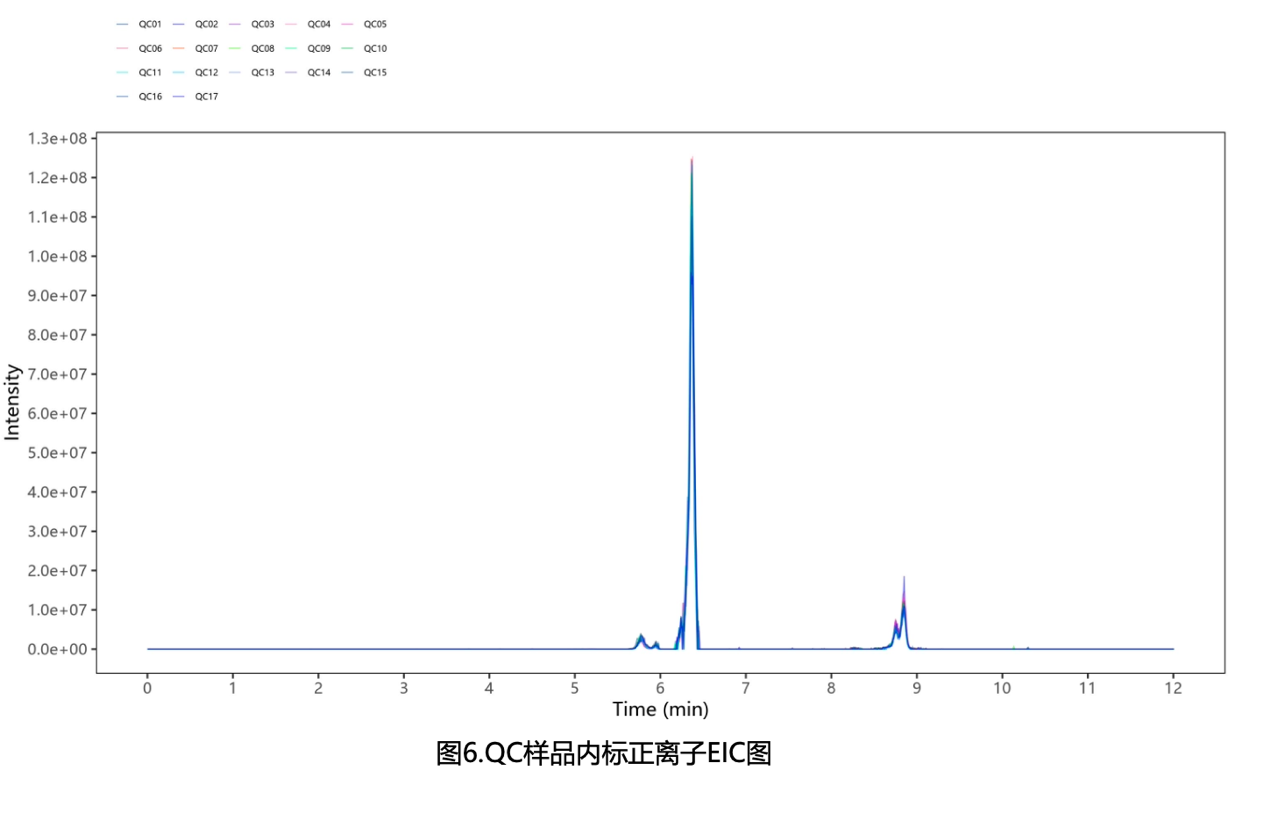


**Extracted ion chromatograms (EIC) of internal standards in QC samples in positive ion mode.**


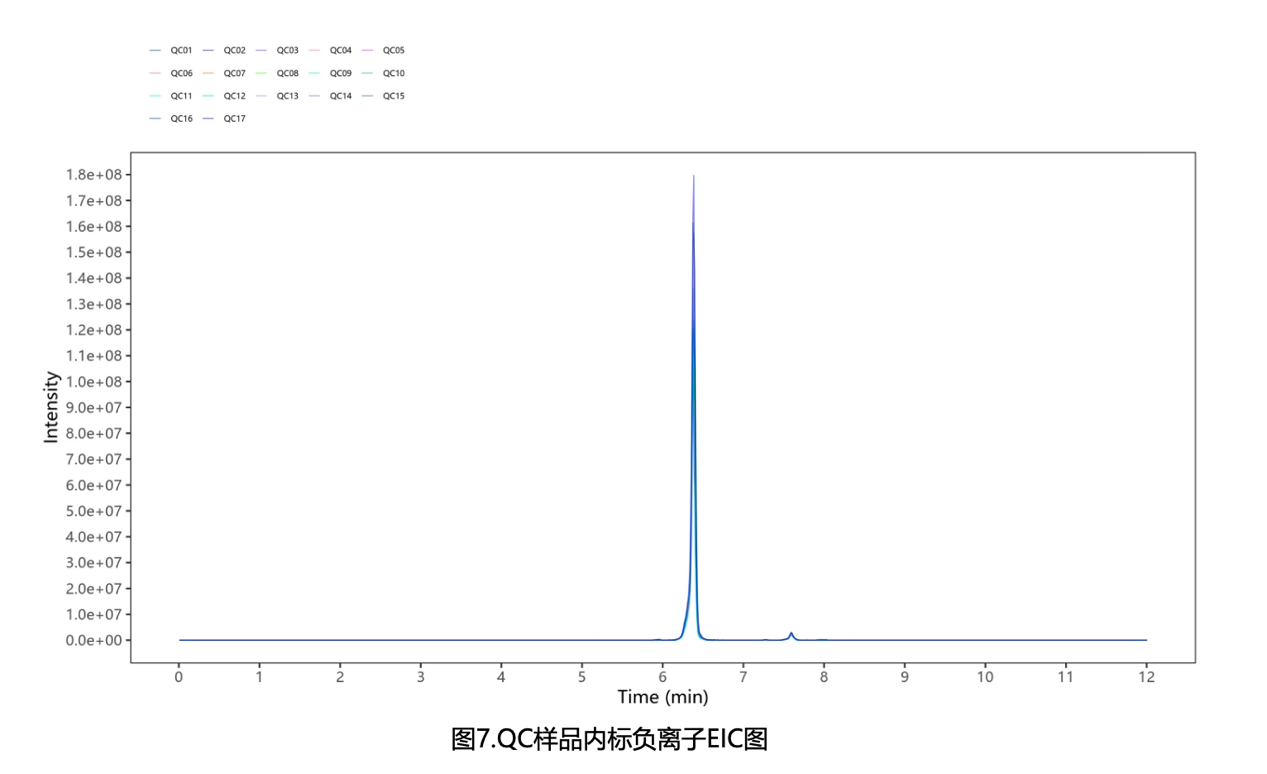


**Extracted ion chromatograms (EIC) of internal standards in QC samples in negative ion mode.**


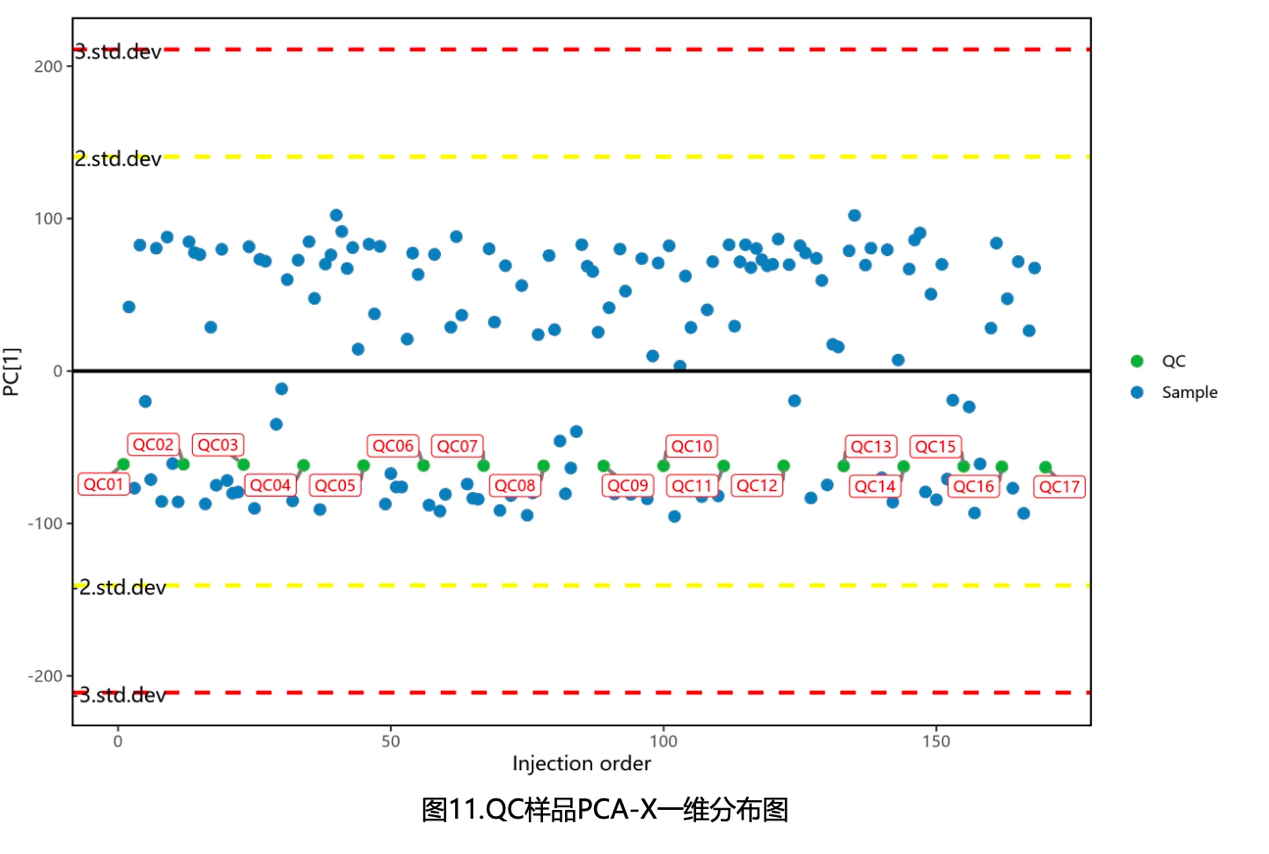


**One-dimensional PCA score plot of QC samples**


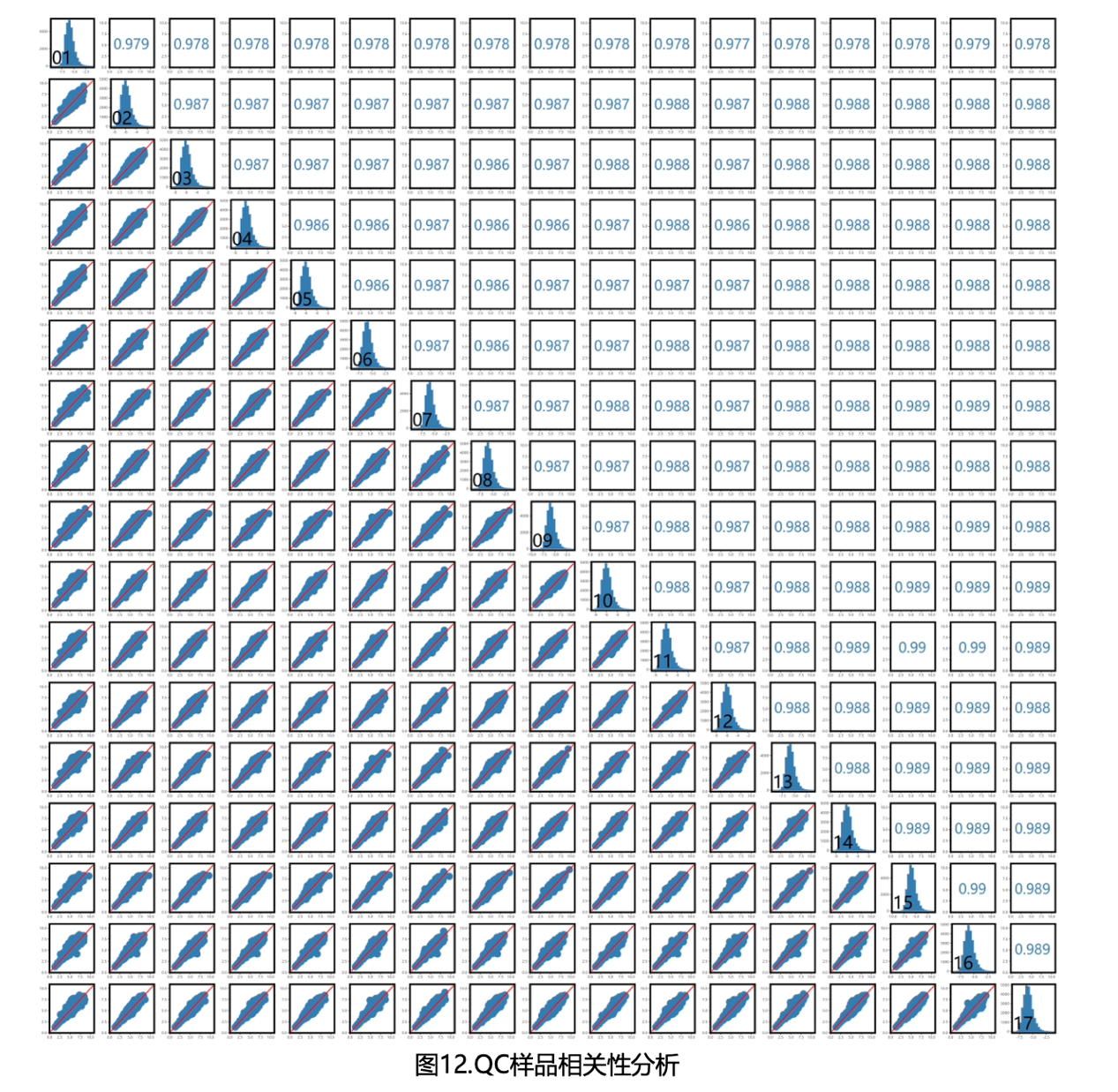


**Correlation analysis among quality control (QC) samples**


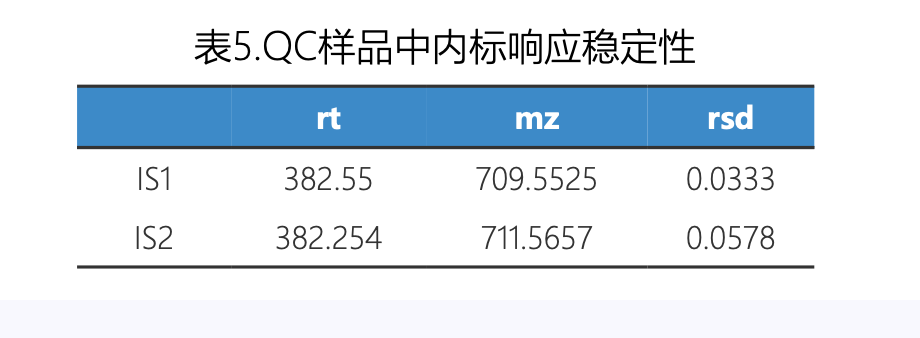


**Internal standard response stability in QC samples**
