# Supplementary figures and images for: Dapagliflozin induces renal lipidomic remodeling and systemic metabolic improvement
Source: Biol Direct. 2026 Apr 17;21:50. doi: 10.1186/s13062-026-00800-9 (PMC13094247; doi:10.1186/s13062-026-00800-9)

figure 5C

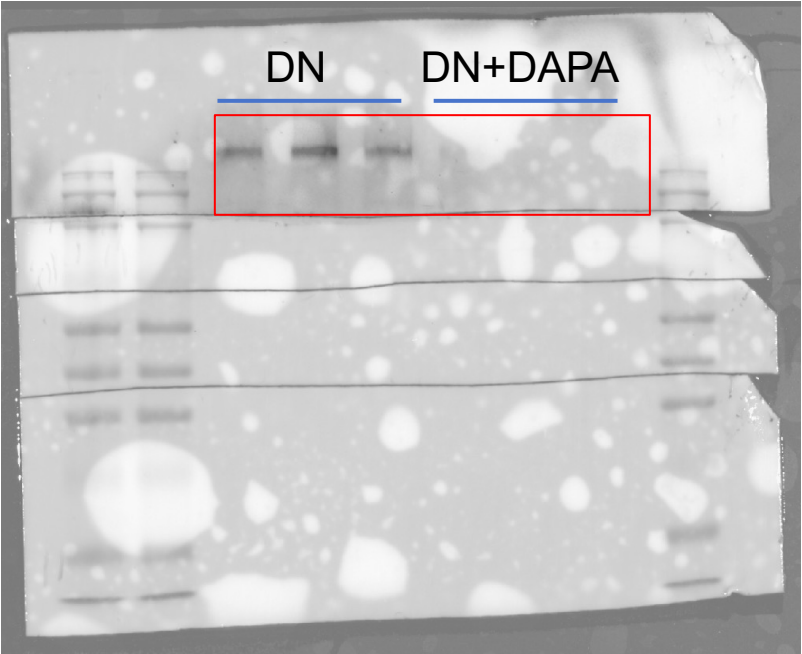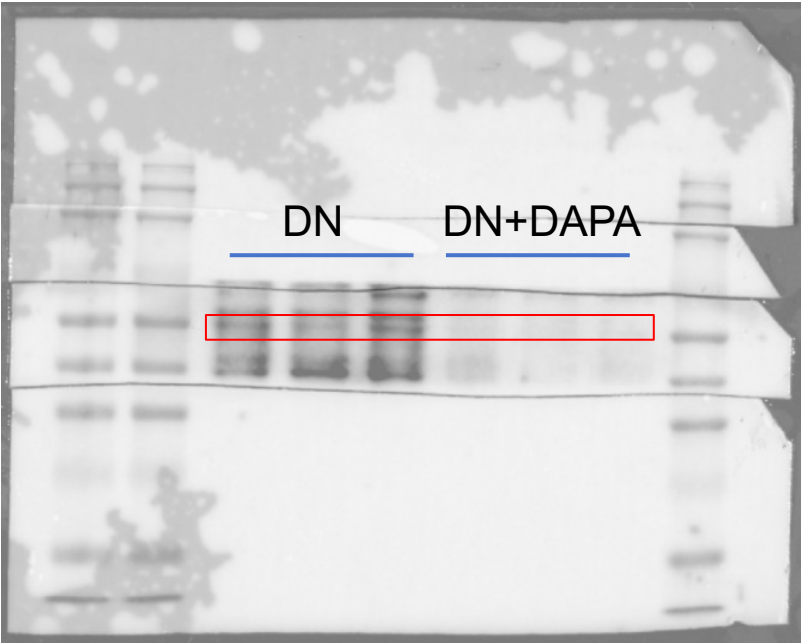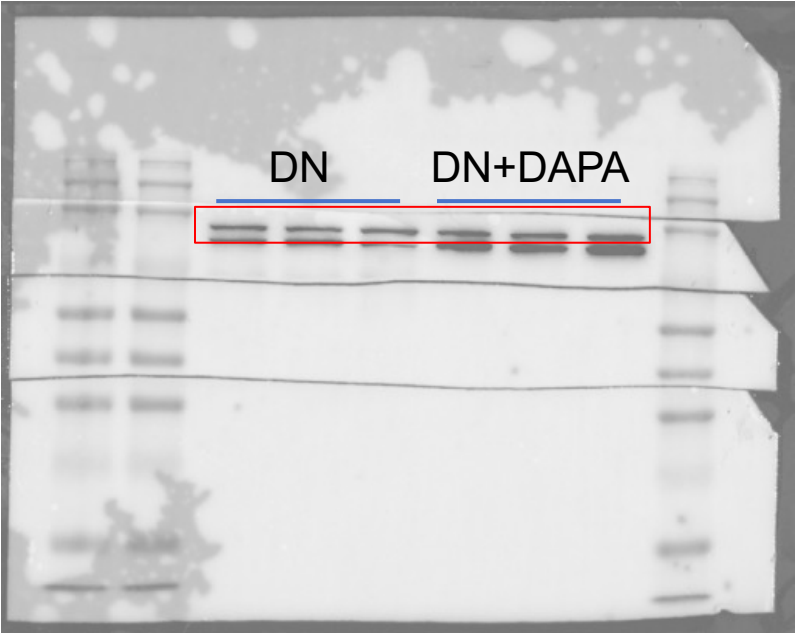

**ACC**

**GPAT3**

**HSP90**

figure 5C

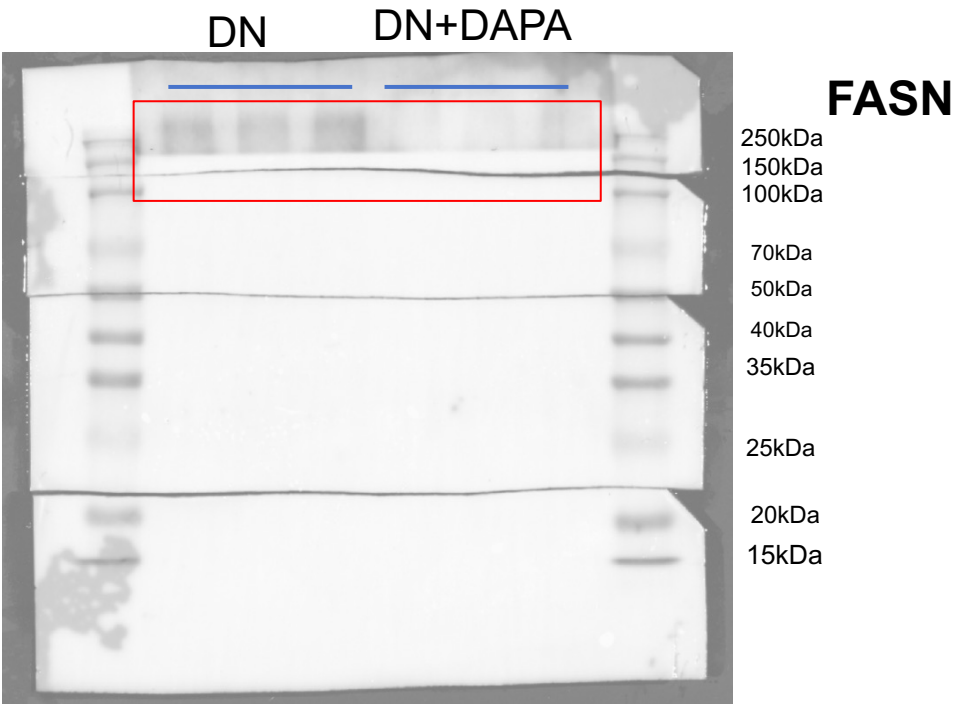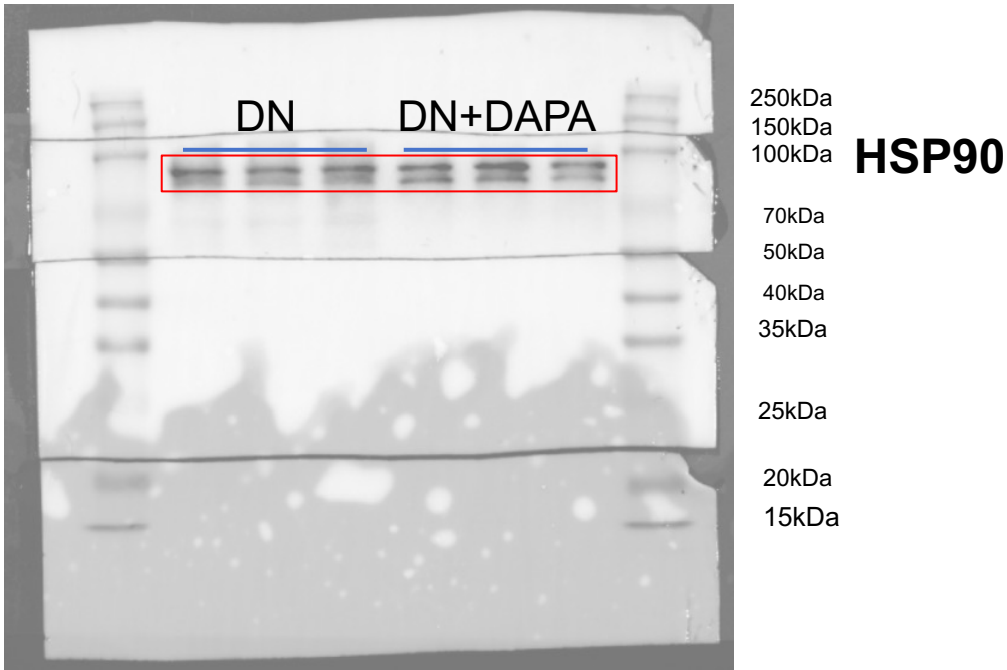

figure 5C

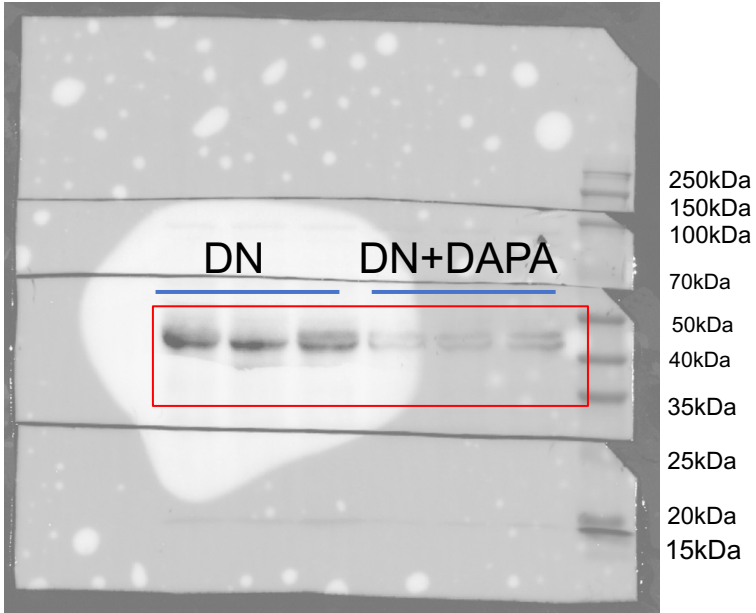

SCD1

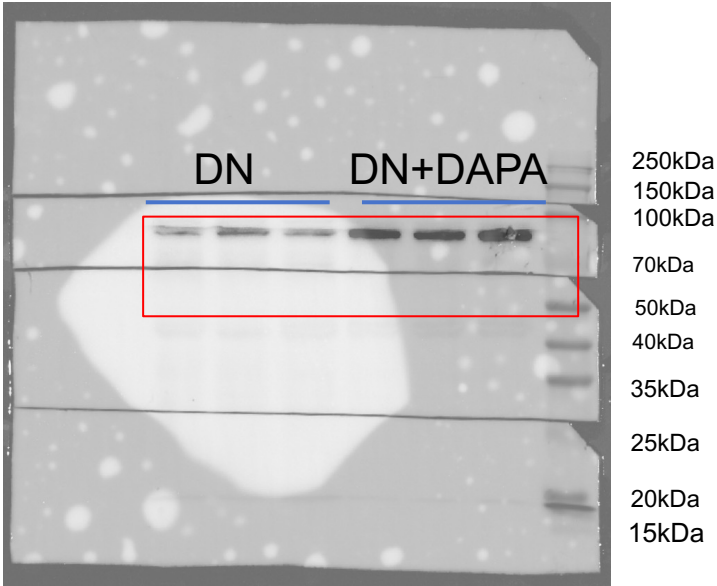

CPT1A

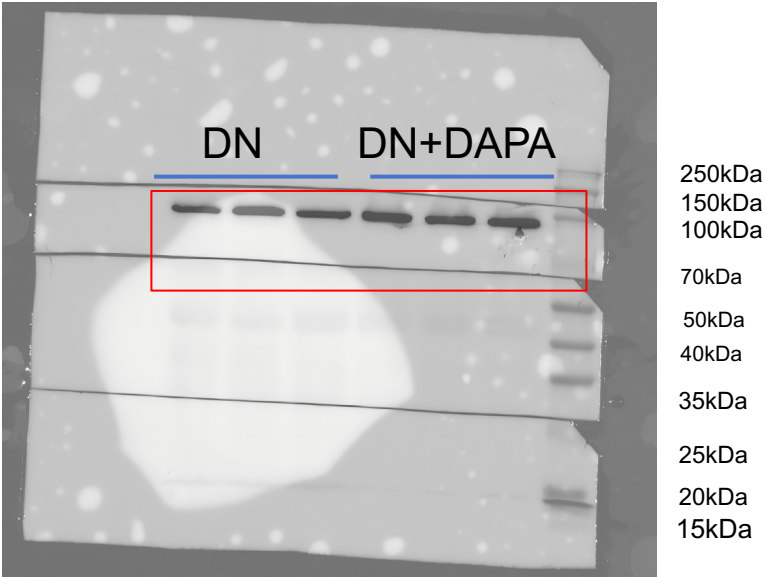

HSP90

figure 6C

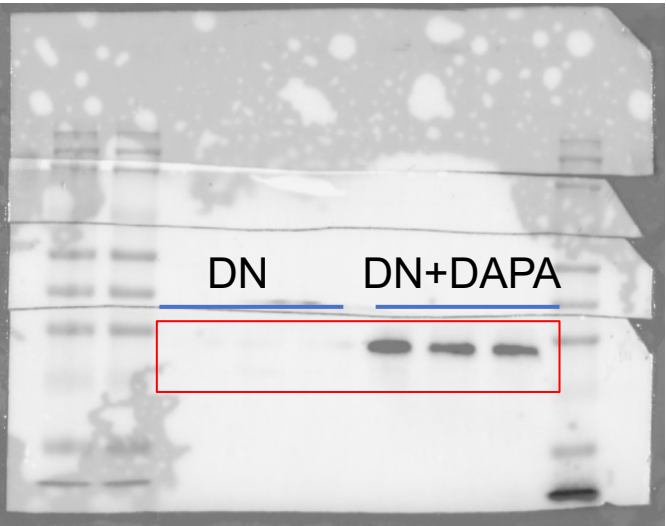

GBA

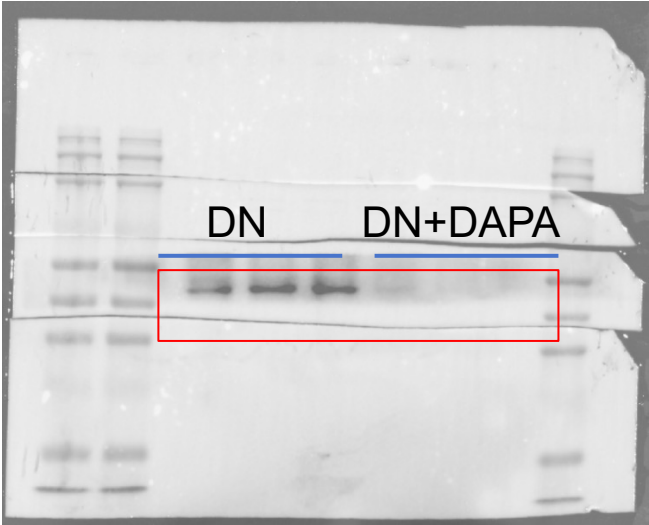

SPHK1

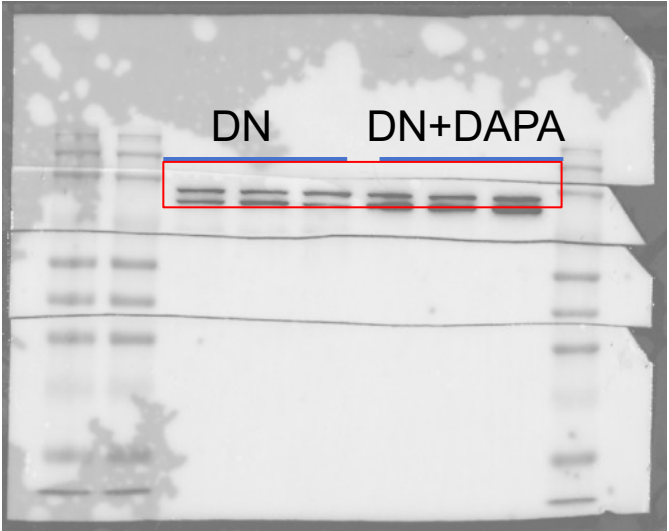

HSP90

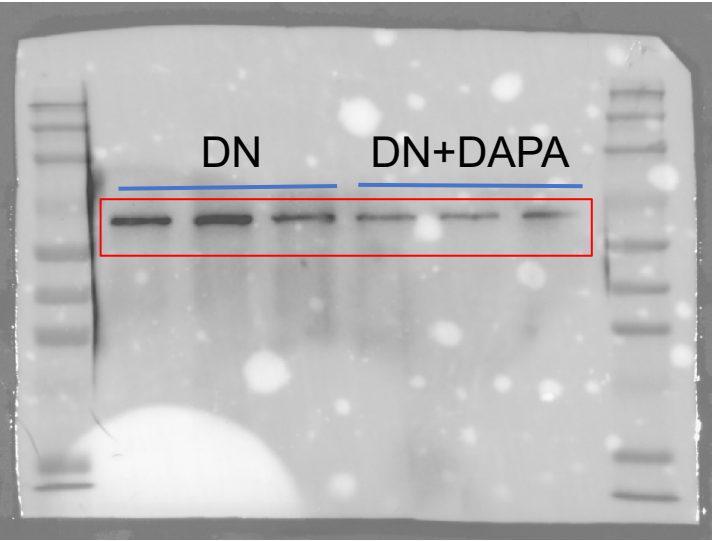

ASM

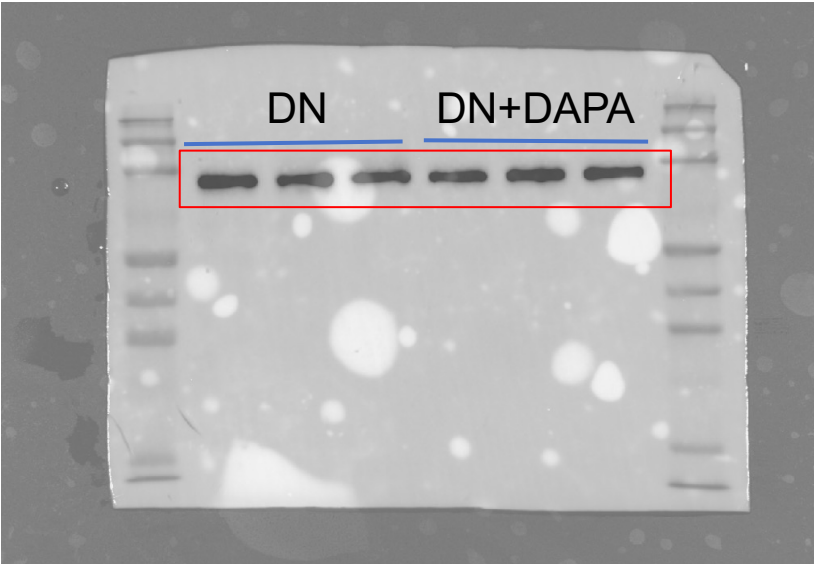

HSP90

figure 7C

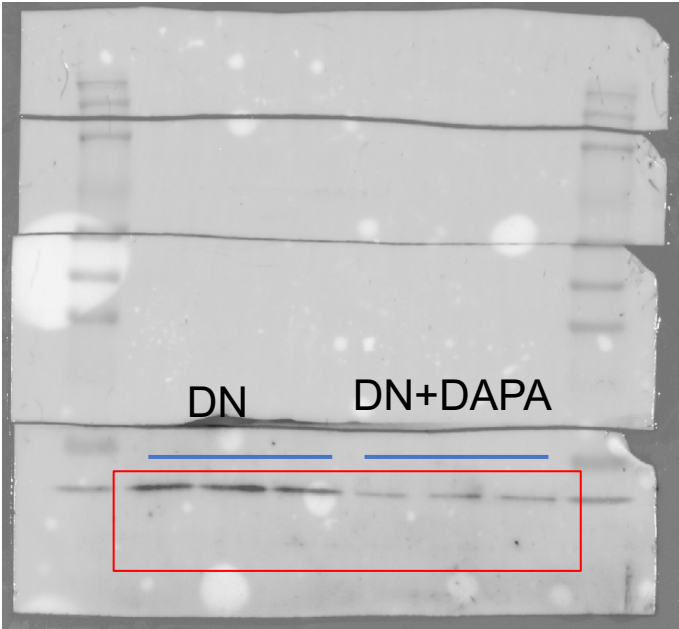

PLA2G1B

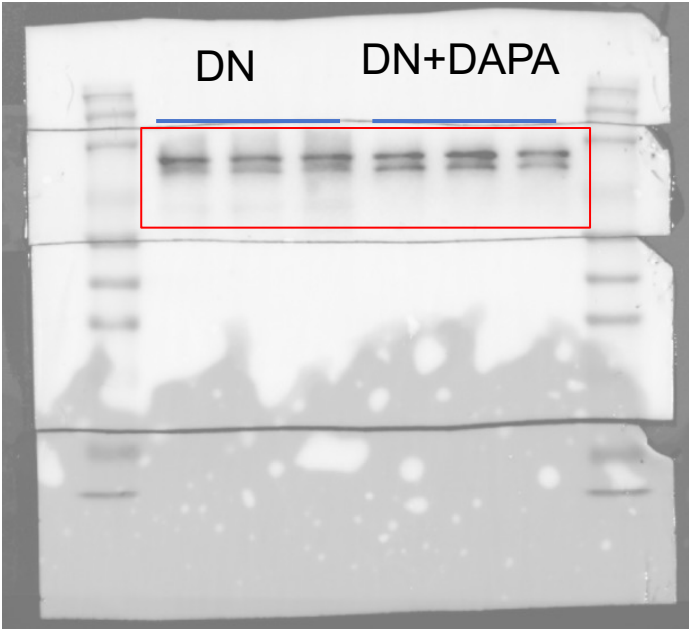

HSP90

Supplement: Supplementary file 4 — Supplementary Material 4 [file 13062_2026_800_MOESM4_ESM.pdf]
